# Integration of geophysical and remote sensing data for structural analysis and delineation of gold, fluorite, and barite mineralization in the Dawi shear belt, Egypt

**DOI:** 10.1038/s41598-026-52061-1

**Published:** 2026-06-11

**Authors:** Ahmed M. Eldosouky, Mohamed A. Abd El‑Wahed, Mohamed Attia, Reda A. Y. El-Qassas, Mayada A. Eldeeb, Mahmoud Atef, Nevin Aly

**Affiliations:** 1 Department of Geology, Faculty of Science, Suez University, P.O. Box: 43221, Suez, Egypt; 2 Geological Engineering and Geophysics Department, Faculty of Petroleum and Mining Engineering, Suez University, P.O. Box: 43221, Suez, Egypt; 3 Geology of Petroleum and Natural Gas Program, Faculty of Science, Suez National University (SNU), P.O. Box: 43221, Suez, Egypt; 4 Geology Department, Faculty of Science, Tanta University, P.O. Box: 31527, Tanta, Egypt; 5 State Key Laboratory of Continental Evolution and Early Life, Department of Geology, Northwest University, Xi’an, 710069 China; 6 Geology Department, Faculty of Science, Kafr El Sheikh University, P.O. Box 33511, Kafr El Sheikh, Egypt; 7 Ground Geophysics Department, Exploration Division, Nuclear Materials Authority, P.O. Box: 530, Maadi, Cairo Egypt; 8 Geology Department, Faculty of Science, Sohag University, Sohag, Egypt

**Keywords:** Egyptian Nubian Shield, Dawi shear belt, Aeromagnetic data, Synthetic aperture radar, Multisource remote sensing, Gold and fluorite, Environmental sciences, Solid Earth sciences

## Abstract

This study integrates multispectral satellite imagery, aperture radar (SAR) data, aeromagnetic data, and field observations to identify and delineate hydrothermal alteration zones associated with gold, fluorite, and barite deposits. This integration complements lithologic and structural mapping of the Dawi shear belt within the East African Orogenic Belt. The Enhanced Horizontal Gradient Amplitude (EHGA) filter was applied to RTP aeromagnetic and upward-continued (UWC) data at altitudes of 0.5, 1, and 2 km. The data delineated shallow and deep structural trends that may control mineralized zones of gold, fluorite, and barite. Euler deconvolution and source-parameter imaging were used together to reveal changes in the basement surface. The Dawi shear belt has experienced multiple deformation phases, beginning with NNW-SW shortening, followed by ENE-WSW compressional and sinistral transpression. This deformation is driven by NW-SE-oriented Najd shearing, which creates N-S-trending folds and dextral shearing, culminating in NE-trending folds. Sentinel-1 A backscatter images were used to enhance structural mapping and lineament detection, which are important for hydrothermal ore deposits and gold mineralization. PCA-based lineament density mapping from S1-A data revealed medium to moderately high concentrations in gneissic, ophiolitic, sedimentary, and volcanic units. The area is divided into three prospective zones for ore exploration, where minerals such as gold and fluorite are likely to be found. Fluorite deposits, classified as vein-type mineralization resulting from hydrothermal fluids, are located within pegmatitic veins aligned NE-SW and NW-SE, crossing schists and metavolcanics. Several zones of gold mineralization occur in the Dawi shear belt, with quartz and quartz-carbonate veins aligned with the Najd Fault System, and gold grades ranging from 0.003 to 0.6 g/t.

## Introduction

**Fig. 1:**
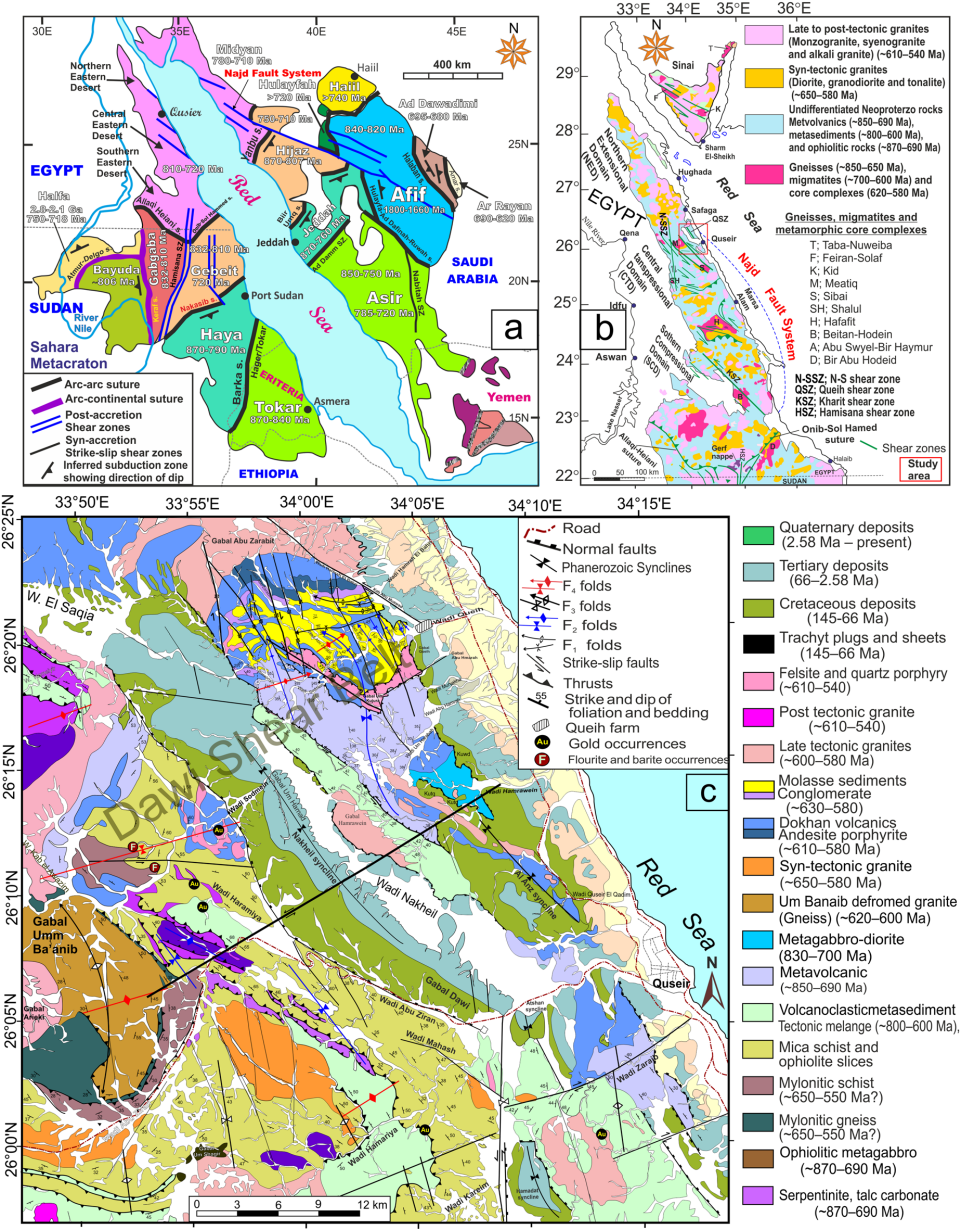
(**a**) Tectonic map of the Arabian–Nubian Shield showing the locations and extents of terranes, sutures and post-accretionary structures (Modified after^13–15^). (**b**) Simplified geological map of the Eastern Desert of Egypt and Sinai showing the Najd fault zone and major structures in the Egyptian Nubian Shield (modified from^1,3,16^), (**c**) Geological map of Dawi Shear Belt (modified and compiled after^17–21^. Available ages from^4,9,13,22–26^. The figures were created by ArcGIS Desktop v. 10.8. https://www.esri.com/en-us/arcgis/products/arcgis-desktop/overview, and SmartSketch v. 4.0 software; https://smartsketch.software.informer.com/4.0/).

Despite numerous geological investigations, the structural controls and spatial distribution of gold, fluorite, and barite mineralization in the Dawi shear belt remain debated. Key questions persist regarding the geometry and continuity of ore-bearing structures, the interplay between surface and subsurface features, and the precise tectonic processes responsible for mineral localization. The Dawi shear belt, which marks the northern boundary of the Najd Fault System (Fig. 1a and b) within the Egyptian Nubian Shield^1–3^ and lies within the resource-rich Golden Triangle district, presents both a geological challenge and an opportunity for mineral discovery. The area affected by the NFS is bounded to the south by the Kharit shear zone and to the north by the Dawi shear belt^4,5^. Although many studies suggest that an initial dextral phase may have preceded the later sinistral movement, the main displacement along the NFS is sinistral^6,7^. The Egyptian Nubian Shield (ENS) is divided into three main structural domains^4^(Fig. 1b): the Northern Extensional Domain (NED), the Central Transpressional Domain (CTD), and the Southern Compressional Domain (SCD). The CTD, the region most influenced by the NFS, exhibits a NW-striking tectonic fabric with transpressional sinistral shearing associated with the NFS. Core complexes were exhumed during orogen-parallel extension around 620–580 Ma, driven by lateral extension and oblique-convergence transpression^3–6,8–12^ along the NFS.

This study integrates multi-source geospatial and geophysical data to effectively unravel the structural architecture, alteration patterns, and mineralization potential of the Dawi shear belt, with a particular focus on gold, fluorite, and barite deposits. Airborne geophysical data are crucial for mapping shear zones, faults, deep structures, and related deposits^10,11,27–31^. Essa et al.^28^. analyzed aeromagnetic and radiometric data to identify and map structural features and mineralization zones, including sulfides, uranium, and gold, in Gebel Umm Tineidba, Egypt. Hamimi et al.^32^ employed structural and potential-field data to define large-scale geological surface and subsurface structures in the Egyptian Eastern Desert. Eldosouky et al.^33^ used aeromagnetic data to map the poorly exposed central western Gawler Craton, Australia, and found that the region can be divided into distinct terranes separated by major shear zones. Additionally, recent advancements in aeromagnetic data analysis have introduced new, more accurate filtering techniques for mapping structures and mineralization^7,31,34–45^.

Multispectral satellite imagery, with appropriate spatial and spectral resolution, effectively captures the spectral absorption signatures of alteration minerals in the VNIR and SWIR bands, enabling the mapping and remote detection of hydrothermal alteration mineral zones and associated lithological and structural features. These zones are key indicators of regions with high potential for ore mineralization exploration, thereby demonstrating the usefulness of multi-sensor remote-sensing satellite data for detailed mapping of these areas^7,10,11,40,46–50^.

Conversely, the active microwave remote sensing instrument, Synthetic Aperture Radar (SAR), is a powerful tool for studying structural regimes. SAR can transmit and detect wavelengths from 2.0 to 100 cm, with common operational bands at 2.5–3.8 cm (X-band), 4.0–7.5 cm (C-band), and 15.0–30.0 cm (L-band)^51^. To effectively map structural features important for orogenic gold mineralization, longer wavelengths (L-band) can be advantageous, as they allow deeper radar signal penetration into the Earth’s surface. Equipped with C-band capabilities, the Sentinel-1 satellite can map structural lineaments linked to hydrothermal gold mineralization in both arid and semi-arid areas^7,12,46,52–55^.

This study aims to: (i) develop an integrated workflow that combines multispectral remote sensing, synthetic aperture radar (SAR), aeromagnetic data, and field observations to improve geological and structural mapping of the Dawi shear belt in Egypt; (ii) delineate and characterize the spatial distribution of lithological units, hydrothermal alteration zones, and structurally controlled mineralization related to gold, fluorite, and barite deposits; (iii) identify and map the major and subsidiary structural features—such as shear zones, faults, and folds—that influence fluid migration and ore localization in the study area; (iv) correlate surface remote sensing and subsurface geophysical data to pinpoint high-potential exploration targets and elucidate the tectonic processes governing mineralization; and (v) establish a transferable methodological framework for mineral exploration in structurally complex and poorly exposed Precambrian terranes worldwide.

.

## Geological setting

The region impacted by the Dawi shear belt exhibits a diverse array of Neoproterozoic rock types, including gneisses, ophiolite slices, metavolcanics, volcaniclastic metasediments, Dokhan volcanics, molasse-type sediments, and felsites^5^. These rocks are intruded by syntectonic granites and metagabbro-diorite, as well as by late- to post-tectonic granites (Fig. 1c). The ophiolite slices include serpentinites, metagabbros, and metabasalts.

The Meatiq Gneiss Dome includes the Um Ba’anib Orthogneiss, the deepest layer, with a crystallization age of 630.8 ± 2 Ma. Above it are mylonitic rocks, part of the Abu Fannani Thrust Sheet, consisting of highly mylonitized metasediments and amphibolites^22,50,56^. The dome also contains syn-tectonic diorite lenses dated at 609.0 ± 1.0 Ma and 605.8 ± 0.9 Ma, indicating active tectonics during the Neoproterozoic era.

Ophiolitic rocks generally include a variety of ultramafic and mafic rocks, including serpentinites, peridotites, gabbros, and basalts. These rocks are remnants of oceanic crust that have been thrust onto continental margins during tectonic events. The ophiolitic rocks exhibit significant shear-induced deformation. This deformation often creates mylonitic zones where rocks are intensely sheared and altered. The granite contains amphibolite enclaves and has a crystallization age of 630 Ma^23^. Above the gneissic granite, high-grade metamorphic rocks are found, which are overlain by thrust sheets composed of low-grade greenschist-facies assemblages^9,24^.

The Dawi shear belt (Fig. 1c) has a dismembered ophiolitic mélange with metagabbros, metavolcanics, metaultramafics, and volcaniclastic metasediments, arranged in thrusts, sheets, and fragments within a mélange matrix. Granitoids intruded these rocks during both syn- and post-orogenic phases. The Wadi El Homeiyir ophiolitic suite near the Sodmein and Qift-Quseir road consists of fragments like serpentinites, metabasalts, and mélange rocks. The El Homeiyir and Sodmein suites form an NW-SE-trending elongated ridge, while the Qift-Quseir ophiolite trends E-W, shifting to NW-SE and NE-SW because of folding.

The Wadi Sodmein ophiolite mainly features serpentinites, which are key components of the oceanic subduction system^24,57^. These occur as tectonic sheets and slices within foliated volcanoclastics, metavolcanic rocks, and along thrusts. They show shearing, foliation, and schistosity, often NW-oriented, matching those of the enclosing metavolcanics. Formed under prograde metamorphism, they are mainly antigorite with minor chrysotile and have refractory chemistry, with low Al and CaO, high Mg, and are derived from depleted harzburgitic protoliths^25,57^.

The Qift-Quseir ophiolite (Fawakhir ophiolite) is an important geological formation in Egypt’s Central Eastern Desert. This ophiolite features a variety of rock types, including serpentinized harzburgite, pyroxenite lenses, gabbros, and pillow basalts. Recent studies have divided the ophiolitic rocks along the Qift-Quseir road into two main groups based on their geochemical traits: (i) MORB-like ophiolites and (ii) SSZ ophiolites^58^. Serpentinized peridotites show significant deformation and shearing, with grey to greyish colors on weathered surfaces and dark green on fresh surfaces. They gradually change into talc-carbonate rocks and display moderate to high relief. Chromite lenses can be found within these formations. Serpentinite bodies are enclosed within metasediments, especially along anticlinal folds. Talc-carbonate formations are situated along fault lines and shear zones. Small, elongated remnants are mainly composed of pyroxenites. The ophiolitic metagabbros appear as blocks oriented northwest, set within a mélange matrix and intermingled with other geological units. At outcrop scale, these metagabbros are sometimes thrust over arc metavolcanics. In some areas, they show extensive deformation, resulting in flaser varieties^59,60^. Recent research has provided zircon U-Pb ages for different parts of the Fawakhir ophiolite, indicating ages of around 816 Ma for the gabbros and 742 − 723 Ma for other intrusive rocks^58,61^.

The volcaniclastic metasediments (Fig. 1c) are interspersed with mafic and ultramafic ophiolitic mélange rocks. They display significant deformation, foliation, and folding, along with pencil-like structures that are observable at outcrop scale. These metasediments comprise metamudstones, metagraywackes, metasiltstones, metaconglomerates, and schists.

In the Queih region, the arc metavolcanics manifest as elongated belts in Queih, Zaraib, and Kab el Awazim. These metavolcanics are variably sheared and classified as intermediate- or acidic-type, comprising meta-andesites, metadacites, metarhyodacites, and associated tuffs^62^.

The Dokhan volcanics (Fig. 1c) are characterized by their unconformity over arc metasediments and molasse-type sediments, which show apparent downfaulting against the arc assemblage, molasse-type sediments, or post-tectonic granites. Felsite also intrudes along Wadi Queih. Three geotectonic models have been proposed for the Dokhan volcanics: anorogenic, orogenic, and transitional^26,63,64^. In the Wadi Queih area, the Dokhan volcanics mainly consist of rhyolites, spherulitic rhyolites, and dacites, while rhyodacites and intercalated dacites are less common. Andesitic tuff deposits are often interbedded with lava flow sequences, which are faulted against metavolcanics and volcanoclastic metasediments characterized by significant shearing and foliation. Porphyritic andesites are common in the northern tributaries of Wadi Queih, including Wadi Umm Zarabit. The Dokhan volcanics are under study and are unconformably overlain from the east by Phanerozoic sediments. They appear as a crescent-shaped outcrop along the northern flank of Wadi Sodmein. They are divided into two main units: a lower unit of intermediate volcanic rocks of andesitic composition, and an upper unit consisting of rhyolitic lava flows, tuffs, ignimbrites, and felsite^7,31^. The extrusion of the Dokhan volcanics likely occurred either before or at the same time as the deposition of molasse-type sediments, as suggested by overlapping whole–rock Rb–Sr ages of 610–560 Ma for the Dokhan volcanics and 600–585 Ma for the molasse-type sediments^65–68^.

The molasse-type sediments cover approximately 60 km² in the Queih basin^69^ and consist of immature green and red mudstones, siltstones, polymictic conglomerates, and sandstones^17,31^. These sediments accumulated through fluvial processes and rest unconformably on the Dokhan volcanics, with deposition believed to have occurred between 615 and 580 Ma, although some studies suggest an alternative age of 645 Ma^1^.

The Wadi Queih basin, situated along the eastern edge of the Precambrian formations, features reddish-purple molasse-type sediments characterized by fining-upward conglomerates and alternating wacke and siltstone layers. These sediments stretch about 12.5 km east-west and are separated by steep normal faults^17,70,71^. The northeastern exposure includes wackes mixed with volcanic flows and basal polymictic conglomerates, interlayered with red siltstone layers up to 10 cm thick. In contrast, the southwest exposure displays a sequence of volcanic flows and clastic deposits formed within a half-graben or down-faulted basin^17,72^.

The molasse-type sediments are interspersed throughout various volcanic flows, suggesting a mixing relationship between the rock strata. A fining-upward sequence that illustrates significant depositional cycles and their subcycles is positioned atop the basal conglomerate. Evidence of subaerial deposition, such as mud cracks and raindrop imprints, is observed in the siltstones. Fragments of metavolcanic and post-tectonic pink granite, which are elements of the early post-collisional alkaline granites, are present in the conglomerates^18^.

The Kujura felsites (Fig. 1c) extend approximately 8.0 km in length and reach a thickness of up to 0.5 km. These formations are characterized as extensive, mylonitized, and fine-grained. The substantial thrust sheet, a product of the arc-related metavolcanics, is prominently exposed in the southern area and lies above all other rock groups. This felsite intruded after the extrusion of the Dokhan Volcanics along pre-existing fractures, signifying the extrusive component of the A-type granite magma within the Eastern Desert^73^.

An elongated body of alkali feldspar and granodiorite-monzonite, including formations such as Gabal Um Zarabit, Kab el Awazim, and Gabal Hamrawein (Fig. 1c), is mostly situated within the Dawi shear belt, where it intrudes into molasse-type sediments, volcanoclastic metasediments, and the Dokhan Volcanics.

The sedimentary rocks in the study area range in age from the Cretaceous to the Quaternary. Arranged from the youngest to the oldest, these formations include: Shagra, Umm Gheig, Umm Mahara, Nakheil, Thebes, Esna, Tarwan, Dakhla, Duwi, and Taref Formations. They are found unconformably overlying the Precambrian basement rocks (Fig. 1b). Among these sedimentary formations, the Duwi Formation stands out, consisting of alternating layers of phosphate, oyster limestone, marl, and black shale^74^.

## Materials and methods

### Multispectral dataset and processing

The multispectral dataset was categorized into three types: free-cloud terrain corrected optical data, radar data, and Digital Elevation Model (DEM) data. For the optical data, Landsat-8 (Path 174/Row 042) and ASTER level 1 T (AST_L1T_00311292000084256_20150413061823_98042) scenes (were sourced from Earth Explorer and NASA’s Earth Data (http://earthexplorer.usgs.gov and https://search.earthdata.nasa.gov/), which were used to map lithological, structural, and hydrothermal alteration zones. The radar data, represented by Sentinel-1 A (S1A_IW_GRDH_1SDV_20250208T154743_20250208T154808_057807_0720E2_80E5) and obtained from the Alaska Satellite Facility, along with four scenes of DEM data from the NASA Earth Data Center, were utilized for lineament extraction and hillshade map creation. The complete set of remotely sensed data was processed using various software tools, including Envi 5.3, ArcMap 10.8, PCI Geomatica 2016, RockWork 2016, and CorelDRAW 64-bit, which allowed for stacking and subsetting to define the study area. Additionally, all data were geometrically corrected using the WGS-84 UTM Zone 36 N coordinate system. This comprehensive approach ensured accurate and detailed analysis of the study area.

Landsat-8 has eleven spectral bands, with seven detecting reflected visible-near infrared (VNIR) and short-wave infrared (SWIR) radiation. Band 9 is especially useful for cloud detection, along with two thermal infrared radiation bands (TIRS, B10 and B11). Additionally, the ultra-blue band 1 is important for identifying coastal and aerosol phenomena (Table 1). The spatial resolutions of these bands are 30 m for bands 1 to 7 and 9, 15 m for the panchromatic band 8, and 90 m for TIRS. The ASTER multispectral sensor features a wide spectral range with 14 bands. This includes three VNIR bands with a resolution of 15 m, six SWIR bands at 30 m, and five thermal infrared radiation bands (TIR) with a resolution of 90 m (Table 1). The European radar imaging satellite Sentinel-1 is equipped with synthetic aperture radar (SAR-C) and uses C-band SAR sensors. These sensors provide dual-polarization (co-polarized VV or HH, and cross-polarized VH or HV), operate in an interferometric wide-swath (IW) mode, and have a spatial resolution of 5 × 20 m (Table 2).

The multisensor data processing is divided into two stages: preprocessing and processing. In the preprocessing phase, the Landsat-8 and ASTER datasets were atmospherically corrected using the Internal Average Relative Reflection (IARR) method to eliminate atmospheric influences that could affect the quality of subsequent analyses, such as image classification and mineral identification^75,76^. For the Sentinel-1 A radar data, the enhanced Lee filter was applied to both VH and VV polarizations to minimize speckle while preserving texture information^77,78^. Additionally, the Digital Elevation Model (DEM) was processed using spatial analysis tools (using the Fill tool in ArcGIS) available in ArcMap to correct surface imperfections^79^. This comprehensive approach ensures that the data is adequately prepared for accurate analysis. Overall, the combination of these methods enhances the reliability of the results obtained from the multisensory data.

**Table 1:** The highlighted aspects of the optical data.

Landsat-8				ASTER			
Band (B)	Wavelength (µm)	Resolution (m)	Spectral region	Band (B)	Wavelength (µm)	Resolution (m)	Spectral region
B1	0.435–0.451	30	Coastal	B1	0.52–0.60	15	VNIR
B2	0.452–0.512		Blue	B2	0.63–0.69		
B3	0.533–0.590		Green	B3	0.76–0.86		
B4	0.636–0.673		Red	B4	1.60–1.70	30	SWIR
B5	0.851–0.879		NIR	B5	2.145–2.185		
B6	1.566–1.651		SWIR	B6	2.185–2.225		
B7	2.107–2.294			B7	2.235–2.285		
B8	0.503–0.676	15	Panchromatic	B8	2.295–2.365		
B9	1.363–1.384	30	Cirrus	B9	2.360–2.430		
B10	10.60–11.19	100	TIR	B10	8.125–8.475	90	TIR
B11	11.50–12.51			B11	8.475–8.825		
–	–			B12	8.925–9.275		
–	–			B13	10.25–10.95		
–	–			B14	10.95–11.65		

Near Infrared “NIR”, Short wave Infrared “SWIR”, Thermal Infrared “TIR”, Visible Near Infrared “VNIR.”.

The transformations applied to the optical satellite imagery, which consist of False Color Composite (FCC), Decorrelation stretching (DS), Band Ratios (BRs), and Principal Component Analysis (PCA), have been executed to outline the lithological units, trace the major structural features, and allocated the hydrothermal alteration zones associated by gold and fluorite ores in the area under consideration^80–82^. This is supplemented by a spectral-matching technique known as ‘Spectral Angle Mapper’ (SAM), which was applied to the first nine bands of ASTER data to monitor the spatial distribution of alteration minerals and fluorite and carbonate minerals^53,83–85^. In addition, band mathematics was utilized on the S1A data to create the VH + VV composite, which was subsequently integrated with the two polarizations, VH and VV, into a single layer that was processed using the PCA technique to extract three principal components (PC1, PC2, PC3) for the automatic identification of linear structures^78^. Furthermore, the Landsat-8 imagery was resampled to 15 m using the Gram-Schmidt spectral pan-sharpening module available in ENVI, thereby enhancing the spatial resolution of the Landsat-8 images and facilitating precise data interpretation^86,87^.

**Table 2:** The highlighted aspects of the Sentinel-1 radar data.

AM	SM	IW	EW	WV
Beam Mode	S1 to S6	IW1 to IW3	EW1to EW5	WV1&WV2
Center Frequency	C-band (5.405 GHz)			
Polarization	SP (HH or VV) DP (HH + HV and VV + VH)			
Spatial resolution (range x azimuth) (m)	5 × 5	5 × 20	25 × 100	5 × 20
Bandwidth (Km)	80	250	4	20 × 20
Chirp bandwidth (MHz)	87.6–42.2	56.5–42.8	22.2–10.4	74.5 & 48.2
Incidence angle (deg)	20–43°	30–42°	20–44°	23 & 36.5°

Acquisition mode “AM”, Stripmap “SM”, Interferometric wide swath “IW”, Extra wide swath “EW”, Wave “WV”, Single Polarization “SP”, Dual Polarization “DP”, Degree “deg”.

The FCC provides tonal color variation by reflecting spectral characteristics of lithologic units and highlighting structural features^88,89^. The decorrelation stretch is a technique that stretches image principal components rather than the image itself^90^. Band ratioing (BR) involves dividing the digital number (DN) of one band by another to produce a grayscale image indicating relative band intensities^91–94^. PCA transforms correlated variables into uncorrelated ones through orthogonal transformation^95–97^. The SAM technique measures angles between pixel spectra and reference spectra (Regions of Interest or mineral spectral libraries) to evaluate spectral similarity^83,84^.

### Aeromagnetic dataset and processing

#### Enhanced horizontal gradient amplitude (EHGA)

The EHGA method^98^, is an improved boundary-delineation filter that uses the arcsine of the ratio of the vertical derivative to the total horizontal gradient (THG) of the total horizontal derivative (THD)^99^. The steps involved in the EHGA process are:

1 $$\:\mathrm{E}\mathrm{H}\mathrm{G}\mathrm{A}=\:\mathcal{R}\left(\mathrm{a}\mathrm{s}\mathrm{i}\mathrm{n}\left(\mathrm{k}\left(\frac{\frac{\partial\:\mathrm{T}\mathrm{H}\mathrm{D}}{\partial\:\mathrm{z}}}{\sqrt{{\left(\frac{\partial\:\mathrm{T}\mathrm{H}\mathrm{D}}{\partial\:\mathrm{x}}\right)}^{2}+{\left(\frac{\partial\:\mathrm{T}\mathrm{H}\mathrm{D}}{\partial\:\mathrm{y}}\right)}^{2}+{\left(\frac{\partial\:\mathrm{T}\mathrm{H}\mathrm{D}}{\partial\:\mathrm{z}}\right)}^{2}}}-1\right)+1\right)\right)\:,$$

where $\:\mathcal{R}$ is true number, and $\:\mathrm{k}\ge\:2$ will produce the best result for real data.

#### Euler-deconvolution (Eu-D)

2 $$\:X\frac{\partial\:F}{\partial\:x}+Y\frac{\partial\:F}{\partial\:x}+Z\frac{\partial\:F}{\partial\:x}+\:{\upeta\:}\mathrm{F}\:={X}_{0}\frac{\partial\:F}{\partial\:x}+{Y}_{0}\frac{\partial\:F}{\partial\:x}+{Z}_{0}\frac{\partial\:F}{\partial\:x}+\:{\upeta\:}\mathrm{b}$$

where, (x_0_, y_0_, z_0_) is the source location; $\:\partial\:F/\partial\:x$, $\:\partial\:F/\partial\:y$ and $\:\partial\:F/\partial\:z$ are the field F derivatives measured at (x, y, z); B is the main field; and *N* is the structural index (SI) that characterizes the source geometry^100^. In our present study, we employed SI = 0.5 to delineate contacts, faults, and boundaries.

#### Source parameter imaging (SPI)

The SPI methodology is used for determining the parameters of magnetic sources from magnetic data. This method is characterized by its simple performance, which makes it fast and adequate^101,102^. The SPI method^103^ uses the local wave number of the analytical signal to define the depth of magnetic sources.

3 $$\:K\:(X,Y)=\frac{\left(\frac{{\partial\:}^{2}T}{\partial\:X\partial\:Z}\:\frac{\partial\:T}{\partial\:X}\right)\:+{\left(\frac{{\partial\:}^{2}T}{\partial\:Y\partial\:Z}\:\frac{\partial\:T}{\partial\:Y}\right)}^{1}+\left(\frac{{\partial\:}^{2}T}{{\partial\:}^{2}Z}\:\frac{\partial\:T}{\partial\:X}\right)}{{\left(\frac{\partial\:T}{\partial\:X}\right)}^{2}+{\left(\frac{{\partial\:}^{2}T}{\partial\:y}\right)}^{2}+{\left(\frac{\partial\:T}{\partial\:z}\right)}^{2}}$$

4 $$\mathrm{Depth} = \frac{1}{Kmax}$$

where the local wave-number $\:Kmax$ is the highest depth over the step source.

The model’s basis is an intricate analytical signal that operates on gridded magnetic data to estimate source elements. The SPI approach uses the correlation between the source depth and the apparent field’s local wave number (k). This can be calculated per location in the grid using both vertical and horizontal components, with the depth usually represented as an image^103^. The SPI is primarily used to delineate the edges and depth of a region’s geological and structural architecture^102,104^.

## Results

### Remote sensing data

#### Lithological discrimination via Landsat-8

**Fig. 2:**
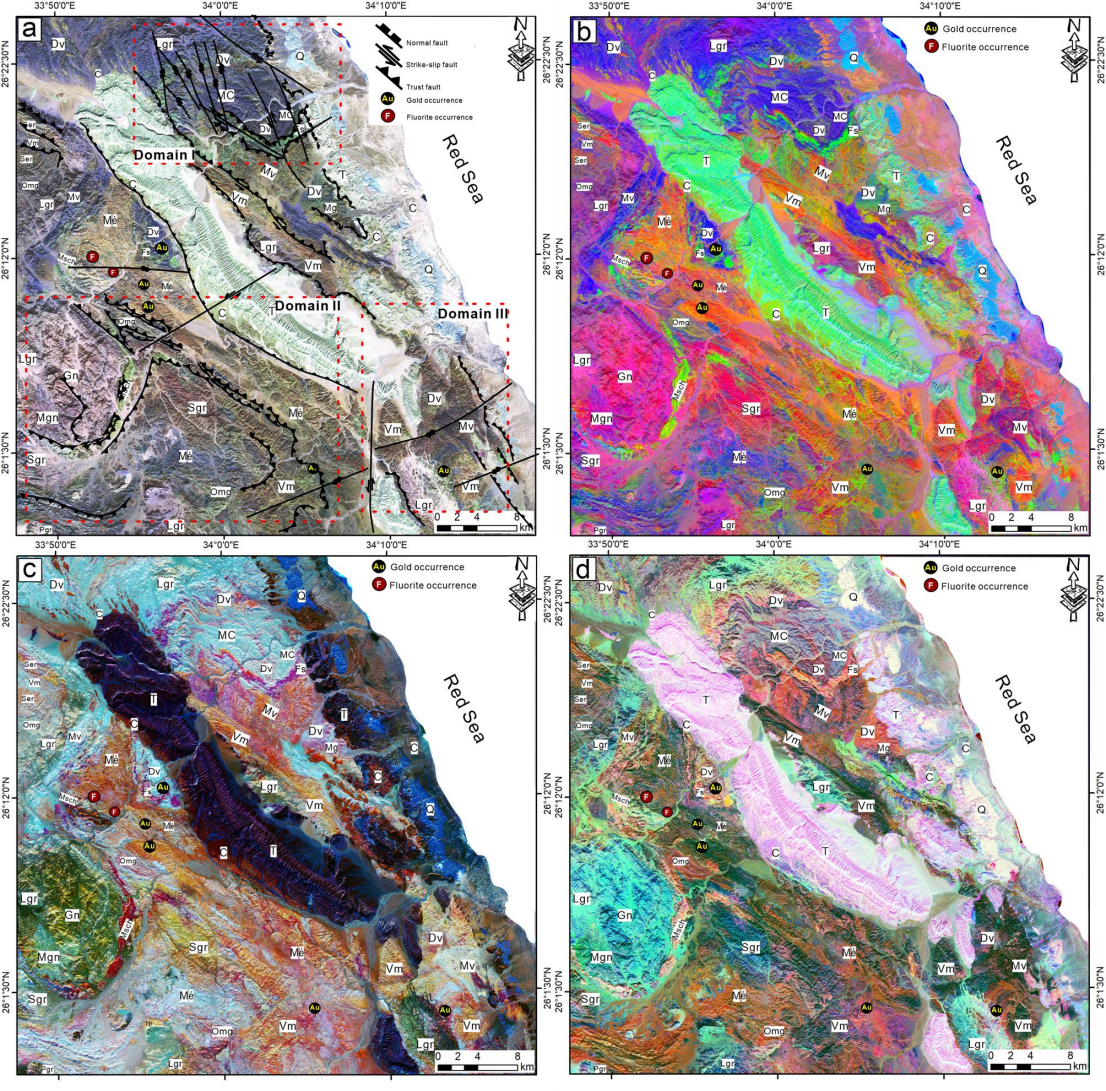
Lithological discrimination utilizing Landsat-8 imgeries in RGB mode; (**a**) FCC765, (**b**) Decorrelated 765, (**c**) BR 2/5 3/6 3/7 and (**d**) BR 6/7 4/3 5/4. Gn=gneiss, Mgn=mylonitic gneiss, Msch=mylonitic schist, Ser=serpentinite, omg=ophiolitic metagabbro, Mé=Ophiolitic mélange, Fs=felsite, Vm=volcanoclastic metasediment, Mv=metavolcanic, Mg=metagabbro, Dv=Dokhan volcanic, MC=molasse sediment and conglomerate, Sgr = syn-granite, Lgr=late-granite, Pgr=poste-granite, Tp=trachyte plug, C, T, Q are Cretaceous, Tertiary and Quaternary deposits. These figures were created and processed by ENVI v. 5.6.2. software: https://www.l3harrisgeospatial.com/Software-Technology/ENVI), which is mainly utilized for image processing, and ArcGIS Desktop 10.8. (https://www.esri.com/en-us/arcgis/products/arcgis-desktop/overview/).

Utilizing the tonal variations of the enhanced Landsat-8 imagery as a key tool to differentiate among the variable lithological units and tracing the valleys of the presented area, the pansharpened color composites Fcc765, decorrelated 765, BRs 2/5 3/6 3/7 and 6/7 4/3 5/4 (Fig. 2a–d) as well as PCs 413 and 214 in RGB mode (Fig. 2a and b) were employed for the lithological discrimination. The Fcc765-RGB (Fig. 2a) highlighted the sedimentary units in pale green, white-green, and pale blue tones for the Cretaceous, Tertiary, and Quaternary units, respectively. In contrast, the basement units, including gneiss, mylonitic gneiss, mylonitic schist, ophiolitic mélange, metavolcanics, volcanoclastic metasediments, Dokhan volcanic, molasse-conglomerates, and felsite, were distinguished by brown, pale violet, lemon, bluish violet, brownish green, yellowish brown, grey, deep blue, and green colors. The granitic magmatic intrusions were marked by reddish brown and greenish brown for the syn and late-tectonic granites.

The decorrelated 765-RGB (Fig. 2b) was utilized to highlight the majority of the lithological borders which the gneiss and mylonitic gneiss marked by deep pink and violet-deep pink respectively and differentiated from the mylonitic schist appeared in shiny yellowish green color, while the ophiolitic sequence including the serpentinite, mélange and metagabbro markable by orange to red orange, violet-blue to violet-orange and pale violet pixels, respectively. The metavolcanics are distinguished from the volcanoclastic metasediments by their orange-green color. In contrast, the Dokhan volcanic and molasse-conglomerate units are distinguished by deep orange to reddish orange, bluish green, and deep blue tones, respectively. Moreover, the sedimentary sequence demarcated in this decorrelated composite by brownish green, shiny apple green, and cyan colors for the Cretaceous, Tertiary, and Quaternary units, respectively. The band ratios of 2/5, 3/6, 3/7, and 6/7, 4/3, 5/4 in RGB effectively emphasize the distinct contacts among the rock units, especially between the gneiss, mylonitic gneiss, and mylonitic schist, as well as among the arc assemblages (e.g., metavolcanic and volcanoclastic metasediment) and the ophiolitic sequence (Fig. 2c and d).

**Fig. 3:**
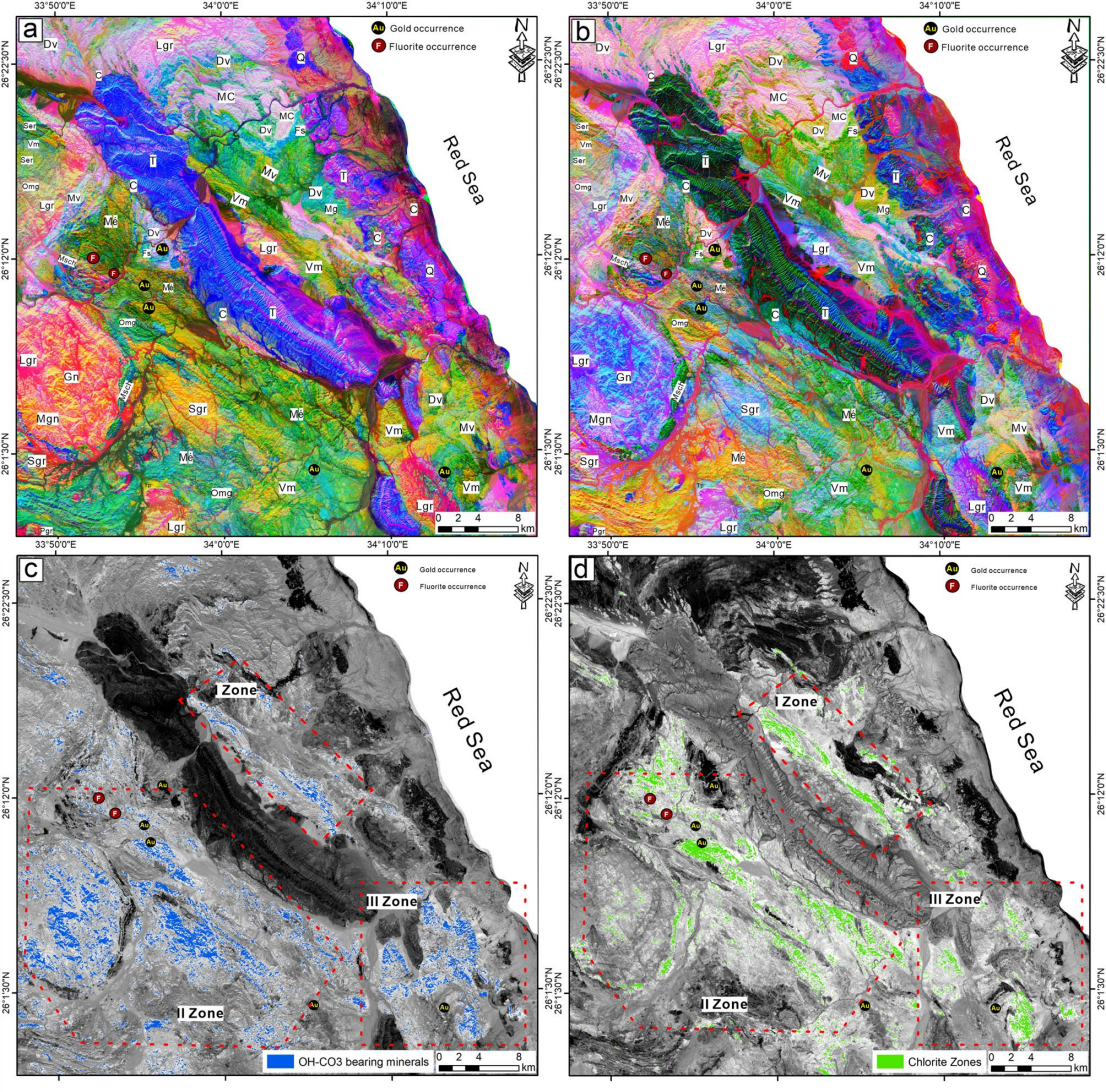
Lithological discrimination utilizing (**a**) PC 413 and (**b**) PC 214 in RGB, of Landsat-8; grey-scaled BRs 7/6 (**c**) and 7/5 (**d**) of Landsat-8 highlighted the OH-CO3 bearing minerals and chlorite zones respectively. For abbreviation see Fig. 2. These figures were created and processed by ENVI v. 5.6.2. software: https://www.l3harrisgeospatial.com/Software-Technology/ENVI), which is mainly utilized for image processing, and ArcGIS Desktop 10.8. (https://www.esri.com/en-us/arcgis/products/arcgis-desktop/overview/).

**Fig. 4:**
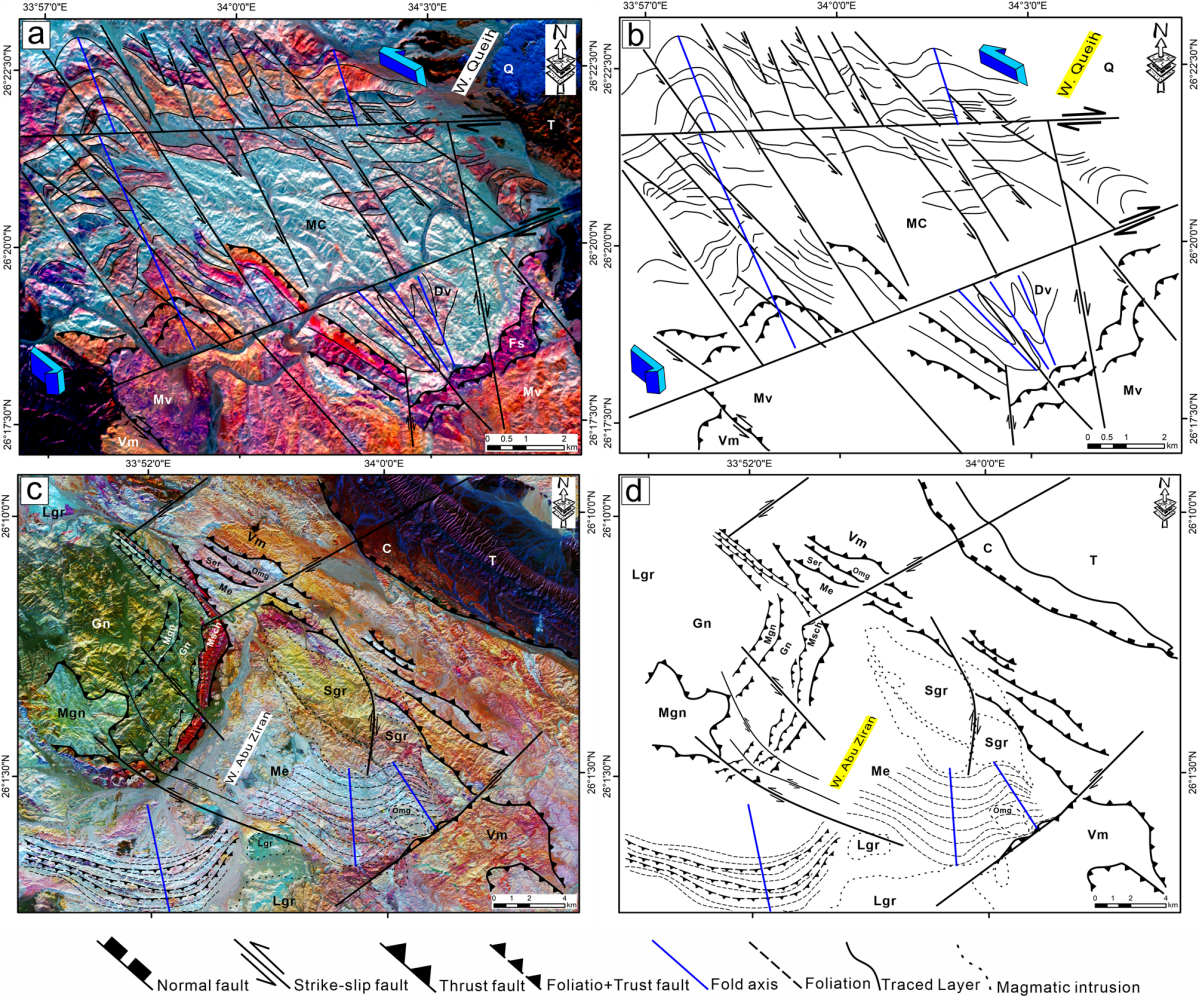
Structural analysis via BR 2/5 3/6 3/7-RGB of Landsat-8; (**a**) and (**b**) Domain I; (**c**) and (**d**) Domain II. For abbreviation, see Fig. 2. These figures were created and processed by ENVI v. 5.6.2. software: https://www.l3harrisgeospatial.com/Software-Technology/ENVI), which is mainly utilized for image processing, and ArcGIS Desktop 10.8. (https://www.esri.com/en-us/arcgis/products/arcgis-desktop/overview/).

Furthermore, both PCs 413 and 214 in RGB (Fig. 3a and b) highlighted the sharp contacts between the magmatic intrusions and the intruded units. In the PC 413, syn, late and post granitic units characterized by orange, pale pink and yellowish pale green (Fig. 2a). In contrast, in PC 214 they were recognized by violet-pale cyan, cyanic pink and lemon tones respectively (Fig. 2b). Both PCs (Fig. 4a and b) were able to highlight the gneissic and mylonitic zones by unique colors from the rest of the surrounding rocks like the ophiolitic sequence, arc assemblages, Dokhan volcanic, felsite and sedimentary sequence.

#### Structural assessment utilizing band ratioing of Landsat-8

Based on structural analyses using the FCC 765 and BR 2/5, 3/6, 3/7 RGB, the investigated area was subdivided into three domains by density of detected structural elements: northeastern, southwestern, and southeastern, in decreasing order (Fig. 2a and c). The BR 2/5 3/6 3/7-RGB has subsetted to outline the northeastern and southwestern sectors, which were regarded as highly deformed (Fig. 4a and c). The tracing of layers and their lithological contacts utilizing the tonal variations displaying the offset among the different rock units, revealed that the northern sector west of W. Queih dissected by three series of strike-slip faults; (i) dominant sinistral NW to NNW, (ii) dextral N-S, and (iii) dextral ENE to E-W strike-slip faults along with the detected thrust contacts striking NW, NE and E-W between felsite, metavolcanics and volcanoclastic metasediment (Fig. 3a and b). Moreover, the traced folded contacts displayed developed folds with fold axes striking NW-NNW (Fig. 4b).

The southwestern sector along W. Abu Ziran is affected by NW-thrust faults separating volcanoclastic metasediments, ophiolitic mélange, and serpentinite. G. Umm Ba’anib features NW to NNW thrusts dividing gneissic rocks and mylonitic zones, along with a major NW-normal fault between sediments and basement rocks. The ophiolitic mélange’s foliation shows folds with axes NW to NNW, cut by sinistral NW strike-slip faults. Offsets suggest northwest-sinistral strike-slip faults dissect Umm Ba’anib gneiss, while northeast-dextral faults intersect central and northern areas. The southeastern zone is dominated by E-W dextral strike-slip faults and NW-normal contacts among Cretaceous, Tertiary, and Quaternary units.

#### Allocation of hydrothermal alteration zones via optical data

The hydrothermal mineral assemblages in altered zones, including clay, carbonate, iron oxides, and minerals rich in Fe and Mg-OH, are mainly associated with altered basic-ultrabasic rocks and display distinct spectral patterns^105^. In contrast, Al–OH mineral groups, such as clay, alunite, and muscovite, are found within altered felsic rocks, and both groups exhibit specific absorption features in the shortwave infrared (SWIR) range. This distinctive spectral behavior allows the use of Landsat-8 and ASTER bands to map these alteration minerals and their related ores^105–107^. Therefore, identifying these minerals is essential for understanding the geological processes and potential ore deposits in the study area.

**Fig. 5:**
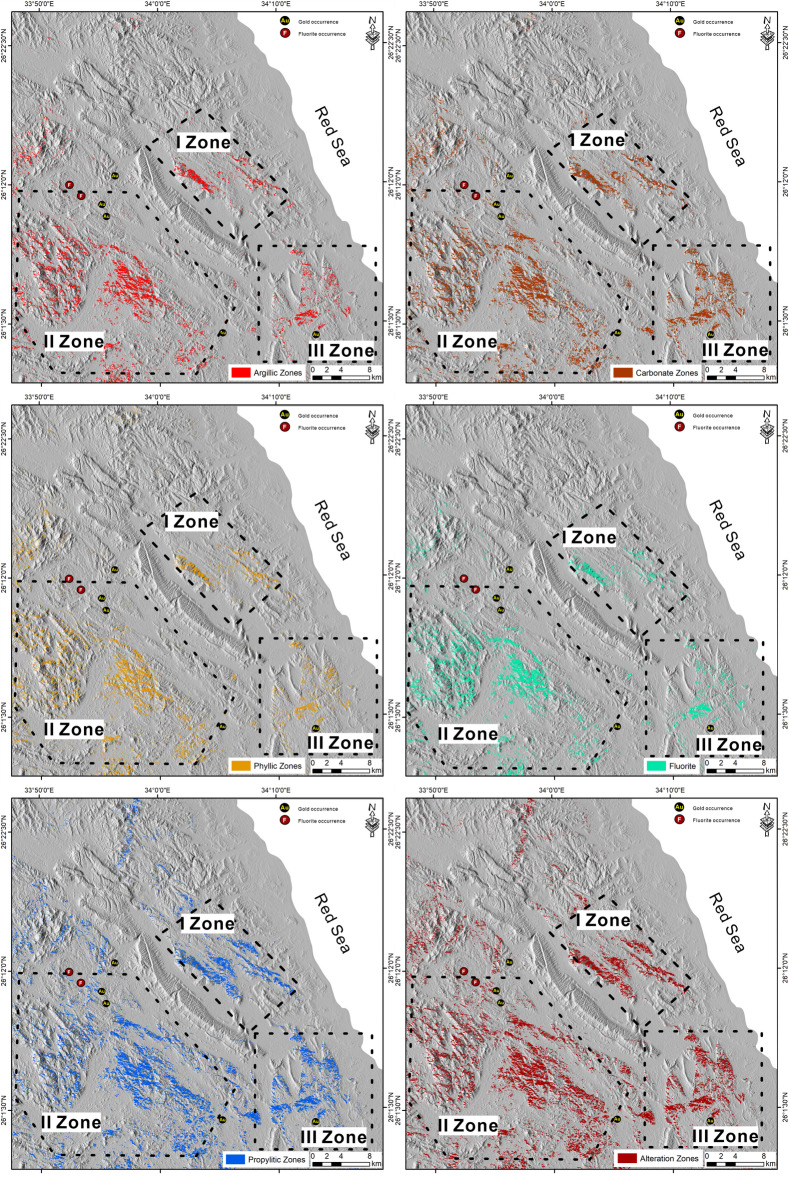
Allocation of alteration and carbonate zones alongside the fluorite minerals via grey-scaled SAM imagery of ASTER, (**a**) Argillic zones, (**b**) Phyllic zones, (**c**) Propylitic zones, (**d**) Carbonate zones, (**e**) Fluorite areas and (**f**) Collective alteration zones. These figures were created and processed by ENVI v. 5.6.2. software: https://www.l3harrisgeospatial.com/Software-Technology/ENVI), which is mainly utilized for image processing, and ArcGIS Desktop 10.8. (https://www.esri.com/en-us/arcgis/products/arcgis-desktop/overview/).

The grey-scaled band ratios 7/6 and 7/5 from Landsat-8 (Fig. 3c and d) were utilized to illustrate the spatial distribution of OH-CO3-bearing minerals and chlorite zones, respectively. Additionally, the Spectral Angle Mapper (SAM) logarithmic technique was applied to the nine ASTER bands to determine the spectral characteristics of the alteration minerals (e.g., kaolinite, montmorillonite, alunite, illite, muscovite, calcite, chlorite, epidote and talc), Fluorite and carbonate minerals through using the spectral library provided by USGS and by moving the rule threshold (Table 3) provided by Envi to allocate the spatial distribution of argillic (kaolinite, montmorillonite, alunite), phyllic (illite, muscovite), propylitic (calcite, chlorite, epidote and talc), fluorite and carbonate zones in the area under consideration (Fig. 5). The surface distribution of alteration zones identified by Landsat-8, including OH-CO3-bearing minerals and chlorite zones, along with those detected by ASTER, such as argillic, phyllic, and propylitic zones, indicates highly concentrated areas that are favorable for gold and fluorite mineralization.

The integration of ASTER-based alteration mapping with Spectral Angle Mapper (SAM) classification successfully identified three distinct mineralized zones characterized by argillic, phyllic, and propylitic alteration assemblages. These zones show strong spatial correlations with known geological structures and are considered highly promising targets for gold and fluorite mineralization. The identified zones include: (i) the southwest sector of G. Dawi along W. Abu Ziran (Zone I); (ii) the southeast sector west of W. Kareim (Zone II); and (iii) the eastern sector along W. Hamrawein (Zone III), as shown in Figs. 3c–d and 5a–c and f.

To specifically map fluorite occurrences, the SAM classification was applied using reference spectra from the USGS spectral library (incorporated within the ENVI software environment). A maximum spectral angle threshold of 0.650 radians was applied to ensure accurate discrimination of fluorite-bearing pixels while minimizing misclassification. This threshold value, along with those applied for other alteration minerals, is summarized in Table 3.

The resulting fluorite distribution map (Fig. 5e) reveals a strong spatial coincidence with the three alteration zones. Notably, the spatial distribution of fluorite and associated carbonate minerals (Fig. 5d) exhibits a distinct concentration hierarchy across the three identified zones. Zone II demonstrates the highest concentrations of both fluorite and carbonate mineralization, followed by Zone III, while Zone I shows relatively lower concentrations of these minerals. This spatial pattern suggests varying intensities of hydrothermal fluid activity across the study area, with Zone II representing the most intensely mineralized sector. The consistent correlation between fluorite enrichment, carbonate occurrence, and the intensity of hydrothermal alteration further supports the exploration potential of these targets, particularly Zone II.

**Table 3:** Summary of the statistical SAM technique.

Alteration Zone	Mineral	Method	Rule threshold	Target count	Average area km^2^
Argillic	Alunite	SAM	0.650	16,910	3324.6511
	Kaolinite			7693	2271.4092
	Montmorillonite			5413	1830.5930
Phyllic	Illite			7016	2315.4539
	Muscovite			7501	2044.1675
Propylitic	Calcite			7165	2191.3398
	Chlorite			10,168	2019.2245
	Epidote			10,020	2149.3562
	Talc			5250	1819.9286
Fluorite	Fluorite			7770	2619.7588
Carbonate	Dolomite			9746	2490.7681
	Magnesite			7624	2153.4070

#### Extraction of Linear structures via S1A-SAR data

Backscatter images generated from Sentinel-1 A, using various off-nadir angles and full polarization, are crucial for enhancing structural mapping and extracting lineaments, which are essential for geological mapping and identifying locations of buried minerals or dilational sites. The rapid extraction of lineaments, particularly fault and fracture zones, is vital because hydrothermal ore deposits are often located near these structures, which facilitate the transport of hydrothermal fluids and gold mineralization^107,108^. In this study, a geometrically corrected Sentinel-1 A image, processed with an adaptive enhanced Lee filter to reduce speckle^109^ and subjected to PCA transformation, yielded principal components that aid in identifying key structural and linear features.

**Fig. 6:**
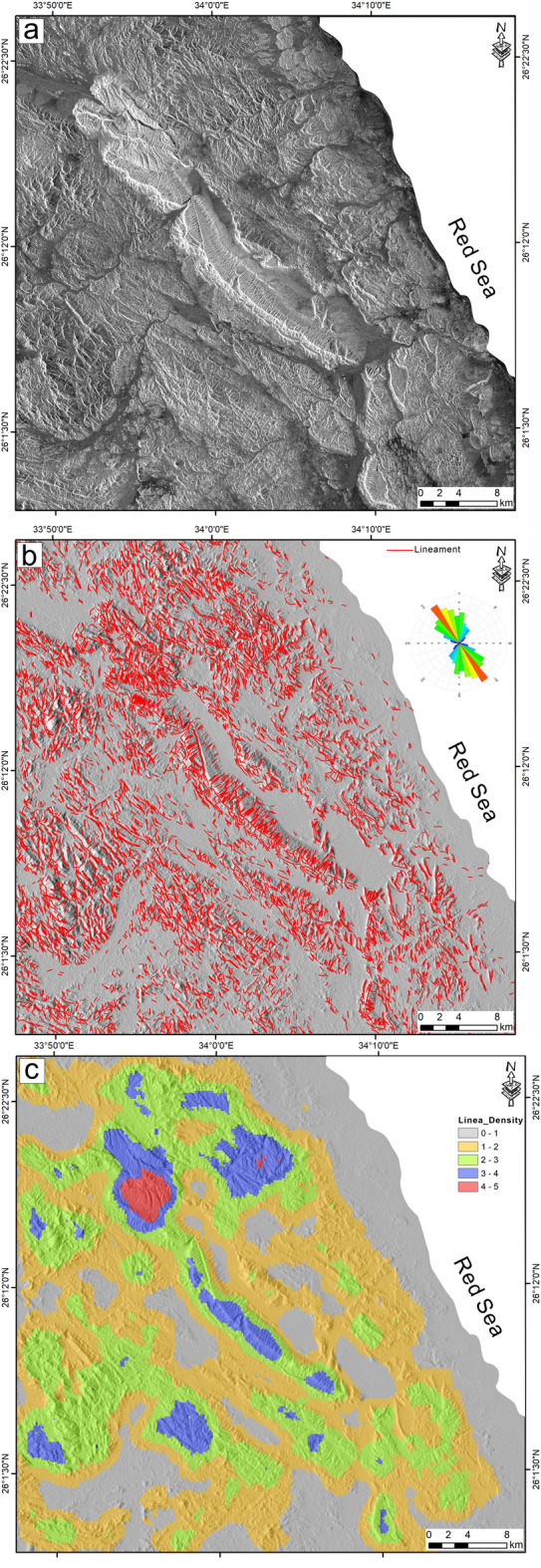
Lineament extraction using S1A SAR data; (**a**) PC1, (**b**) Lineament map and azimuth frequency diagram and (**c**) Lineament density map. These figures were created and processed by ENVI v. 5.6.2. software: https://www.l3harrisgeospatial.com/Software-Technology/ENVI), which is mainly utilized for image processing, and ArcGIS Desktop 10.8. (https://www.esri.com/en-us/arcgis/products/arcgis-desktop/overview/).

The principal component PC1 (Fig. 6a) derived from Sentinel-1 A contains the most critical information on linear structures in the study area. It is processed using the LINE algorithm in PCI Geomatica to facilitate automatic extraction of lineaments. This extraction process employs specific parameter values, including a filter radius (RADI) of 10, an edge gradient threshold (GTHR) of 100, a curve length threshold (LTHR) of 30, a line fitting threshold (FTHR) of 3, an angular difference threshold (ATHR) of 15, and a linking distance threshold (DTHR) of 20, which are essential for accurate execution. The application of these parameters ensures the effective identification of geological lineaments, which are crucial for understanding the area’s structural characteristics.

The principal component analysis was employed for the automatic extraction of lineaments, which highlighted their spatial distribution and azimuth frequency within the study area (Fig. 6b). The extracted lineaments were subsequently processed using ArcMap software to create a density map, illustrating the concentration of lineaments across various rock units (Fig. 6c). The azimuth frequency diagram (Fig. 6b) identified five primary trends of the lineaments in the analyzed area, ranked in order of prevalence as NW-SE, NNW-SSE, N-S, NNE-SSW, and NE-SW. The density map indicated medium to moderately high concentrations of lineaments throughout most gneissic and ophiolitic sequences, as well as in arc assemblages, Cretaceous and Tertiary sediments, and certain regions of the Dokhan volcanic and granitic rocks. This comprehensive analysis provides valuable insights into the structural characteristics of the geological formations in the study area. Overall, integrating principal component analysis and density mapping enhances understanding of lineament distribution and its implications for geological processes.

### Aeromagnetic data

**Fig. 7:**
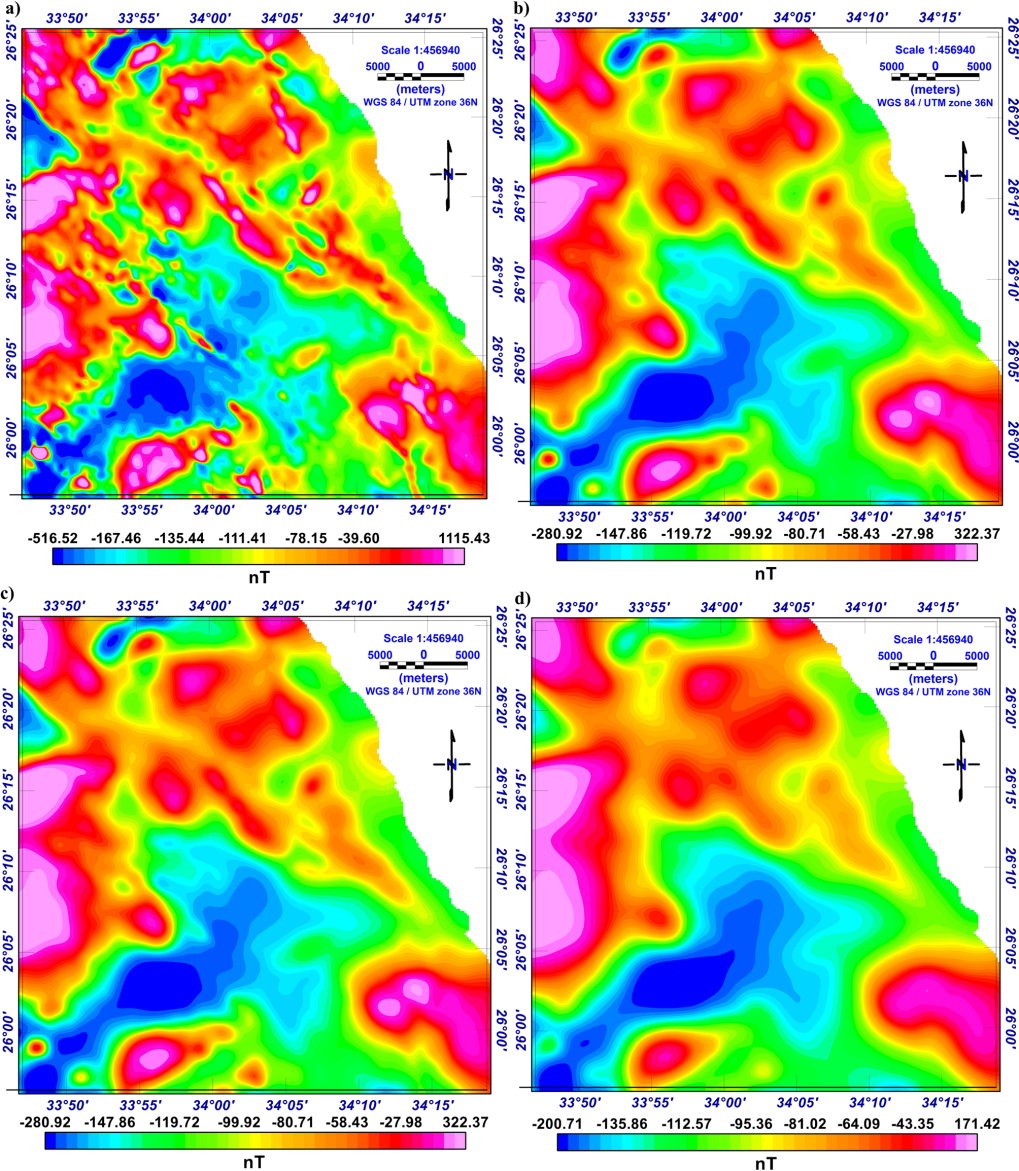
(**a**) RTP map; (**b**) UWC of 0.5 km; (**c**) UWC of 1 km; and (**d**) UWC of 2 km for the Dawi shear-belt area. (The figure was created by Geosoft Oasis montaj v. 8.3.3. https://www.seequent.com/products-solutions/oasis-montaj/).

The reduced-to-north-pole (RTP) data^110^ of the Dawi Shear-Belt area (Fig. 7a) are used to reveal shallow and deep-seated structures. Figure 7 shows magnetic values ranging from − 516.52 to 115.43 nT. Magnetic anomaly trends in NW, NNW, ENE, and NE directions. The RTP data (Fig. 7a) are upward-continued (UWC) to altitudes of 0.5, 1, and 2 km (Fig. 7a–c, respectively).

**Fig. 8:**
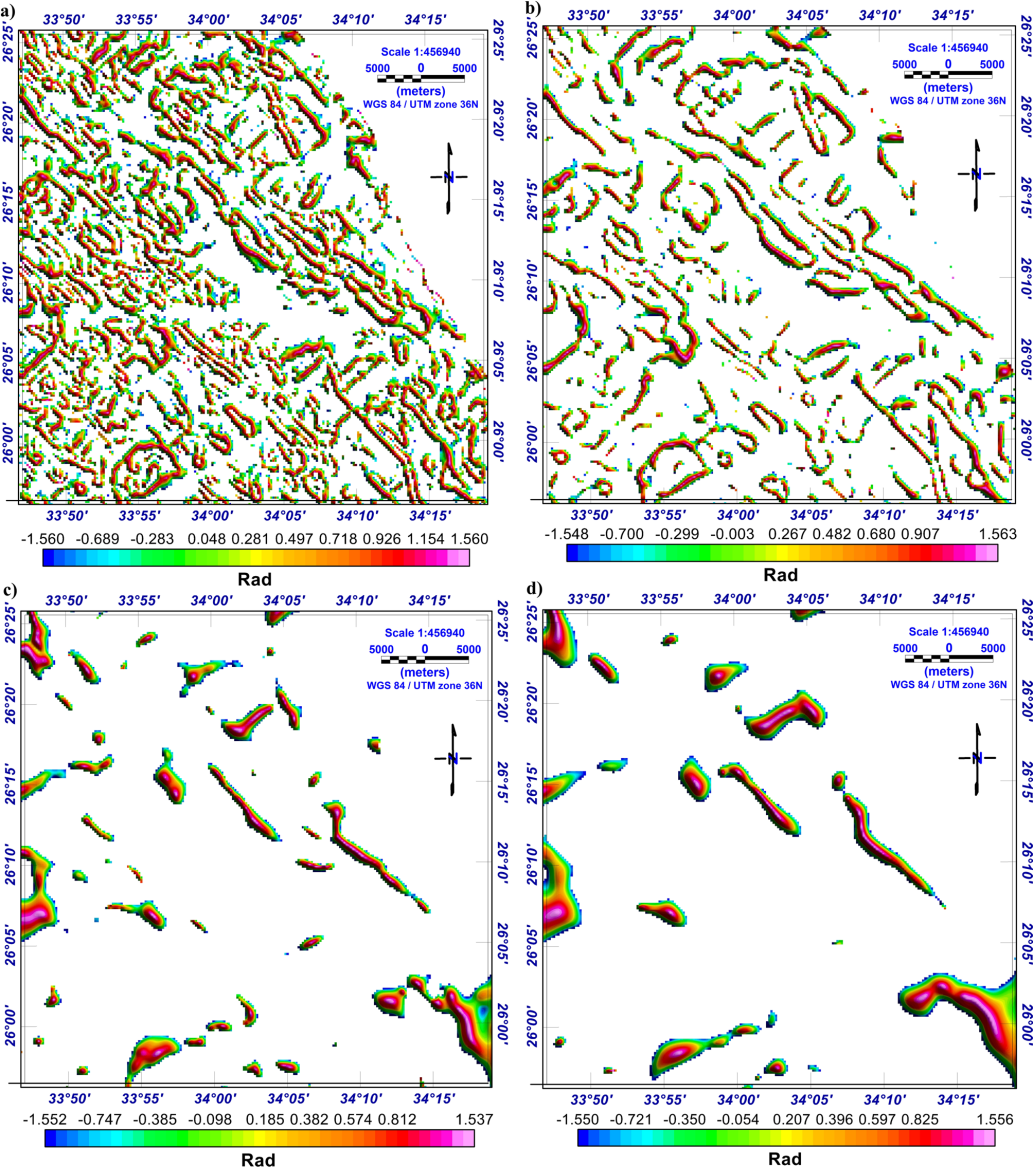
(**a**) EHGA of RTP; (**b**) EHGA of UWC at 0.5 km; (**c**) EHGA of UWC at 1 km; and (**d**) EHGA of UWC at 2 km (the figure was created by Geosoft Oasis montaj v. 8.3.3. https://www.seequent.com/products-solutions/oasis-montaj/).

To reveal the shallow- and deep-seated structures affecting the Dawi shear belt, the EHGA method is applied to RTP and UWC data^7,31,111–113^. The EHGA map of RTP (Fig. 8a) delineates the main structural lineaments controlling the area. The dominant structures are NW-SE, NE-SW, N-S to NNW-SSE, and ENE-WSW, with minor traces of WNW-ESE and E-W. At 0.5 km depth, the EHGA-UWC map (Fig. 8b) shows that NW, NE, WNW, NNW, N-S, and E-W are the dominant structures. At 1 and 2 km (Fig. 8c and d, respectively), NW, ENE, and NE dominate the area.

**Fig. 9:**
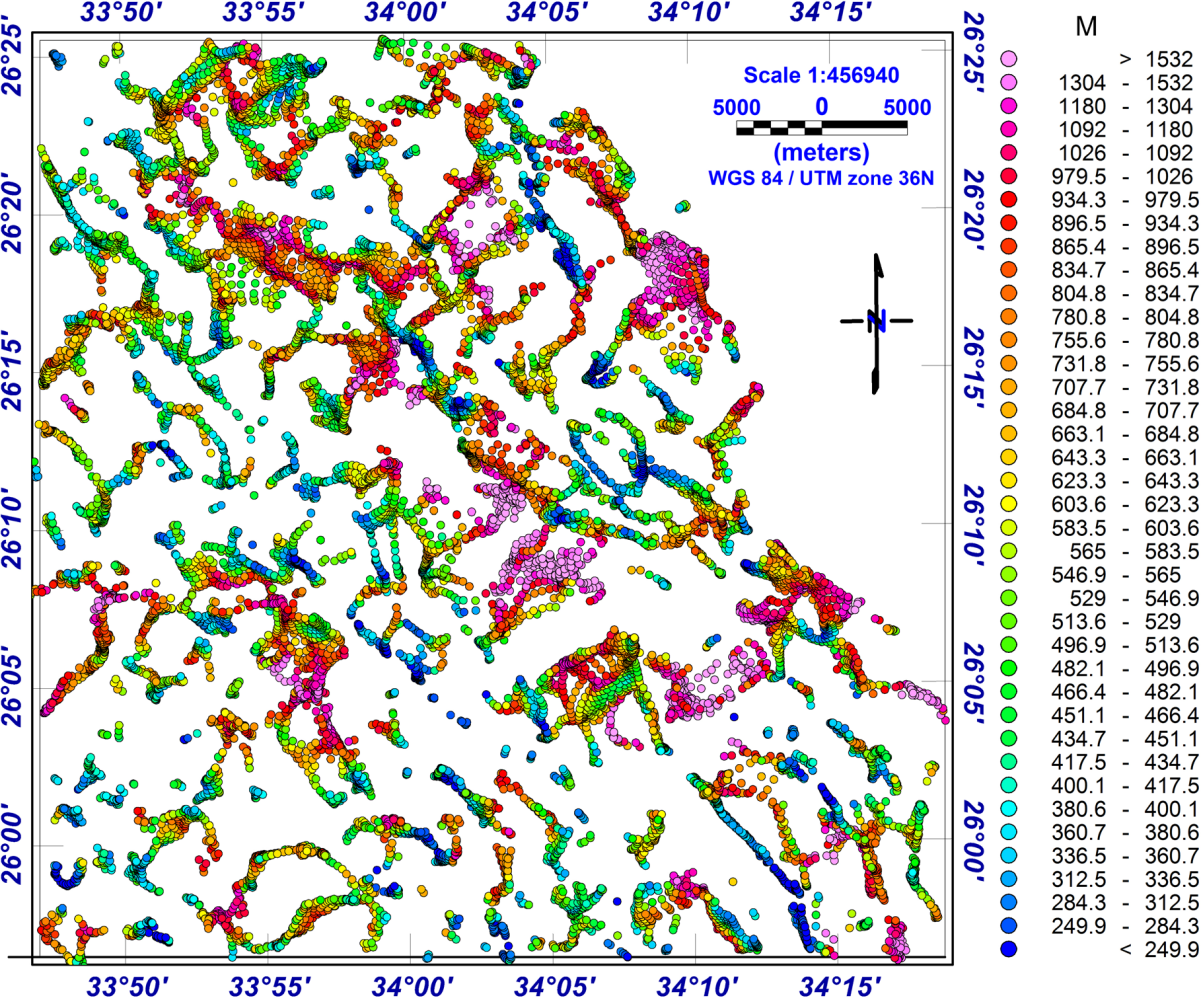
Eu-D map of the Dawi shear-belt area using SI = 0.5. (The figure was created by Geosoft Oasis montaj v. 8.3.3. https://www.seequent.com/products-solutions/oasis-montaj/).

**Fig. 10:**
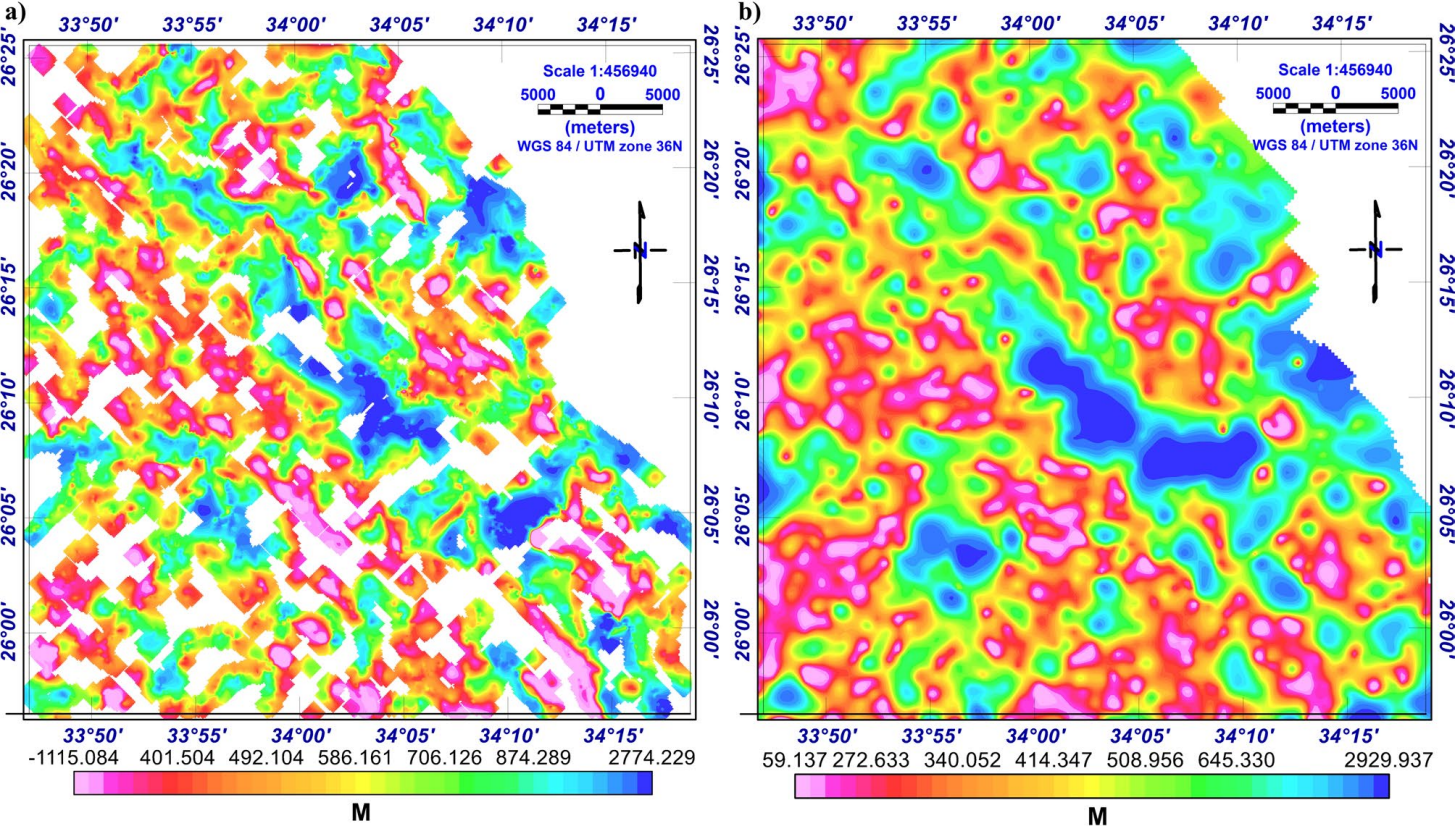
(**a**) Eu-D depth map; and (**b**) SPI map (the figure was created by Geosoft Oasis montaj v. 8.3.3. https://www.seequent.com/products-solutions/oasis-montaj/).

To delineate the depths of structures and basement relief, Eu-D is applied to RTP data employing a SI of 0.5. Figure 9 shows the results for Eu-D, which reveal that contact depths exceed 1.5 km. Moreover, the depths increase under Gabal Duwi. The Eu-D solutions are contoured to produce the Eu-D depth map (Fig. 10a). Figures 6b and 10a show that the structural trends controlling the area are NW, NE, N-S, ENE, WNW, and E-W. Figure 10b shows the results of the SPI method. The depths obtained from Eu-D and SPI (Fig. 10a and b reach 2.7 km and 2.9 km, respectively.

The results of Eu-D and SPI for basement depth help identify deep-seated structural passageways that may direct hydrothermal fluids. Moreover, it can help delineate uplifted blocks and structural depressions that affect fluid concentration. This provides insights into the structural pattern influencing hydrothermal alteration zones^30,114,115^.

### Structural setting

Examining the EHGA filter of RTP, along with remote sensing and field studies conducted in the Dawi shear belt, reveals a complex structural arrangement shaped by notable deformation stages. The Dawi shear belt comprises the Quieh and Abu Ziran NW-trending shear zones, which are divided by Gabal Dawi, forming a graben and two horst blocks.

The ENE-oriented structural networks seem to trace the oldest tectonic structures preserved in the area. This ancient orientation is overlain by a prominent NW-trending structural lineament, which likely indicates a regionally extensive, more recent deformation phase. Moreover, a significant N-S shift is identified, suggesting overprinting or subsequent reactivation events. Although NE-directed features are also observed, they play a lesser role in shaping the overall structural framework. The curvature patterns revealed by the EHGA methodology indicate the presence of folding systems in the Queih and Sodmein regions, where both large-scale synclines and anticlines are evident. The folds show rotation along a northeast-oriented axis, pointing to a folding event driven by compressional forces aligned with the primary structural orientations. These findings suggest a polyphase deformation history and highlight the importance of integrating aeromagnetic geophysical data with geological context to better understand the tectonic evolution of the Dawi shear belt.

The deformation phases within the Dawi shear belt start with NNW–SSE shortening (D1), followed by ENE–WSW compression and sinistral transpression (D2) due to the NW-SE-oriented Najd shearing. This is succeeded by a prolonged E-W compression phase that creates N-S-trending folds (D3) and dextral shearing along NW-SE shear zones, ultimately leading to the formation of NE-trending folds (D4). Late NNW–SSE normal faults and NNW–SSE sinistral strike-slip faults, likely reactivated during Cretaceous and Red Sea rifting (D5), are also part of this process. A summary of the main structural features of these deformation phases in the Dawi shear belt is provided in Table 4.

#### D1: E-W trending structures

The east-west-trending structures are related to an initial phase of north-northwest-to-west-southwest shortening (D1). These formations are poorly preserved and have been completely eroded by later events. The east-west trending structures are found only in the schists and volcaniclastic metasediments south of the Meatiq gneiss complex and in the molasse-type sediments of Wadi Queih.

**Fig. 11:**
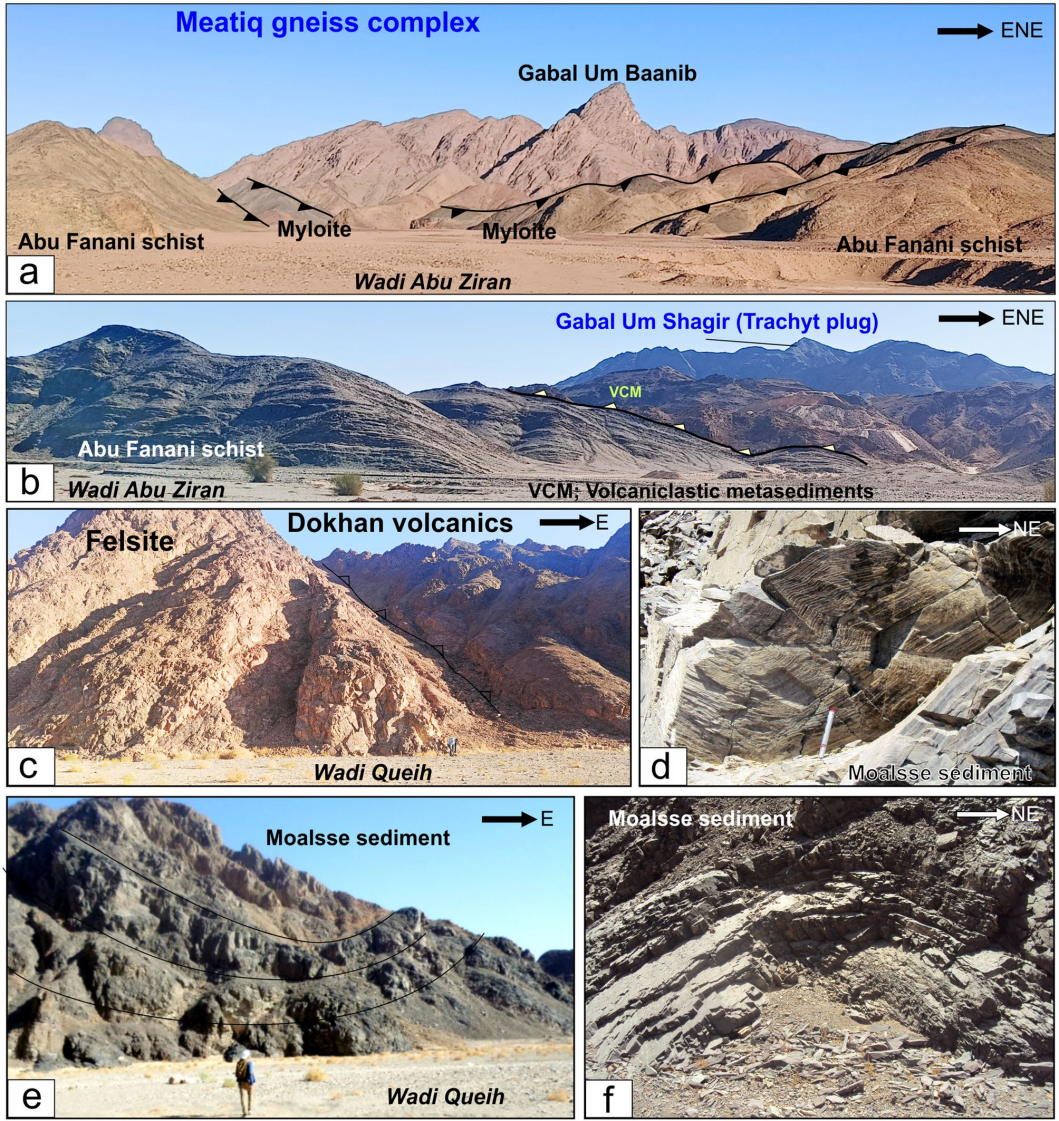
(**a**) Schist and mylonite thrusted over Um Baanib granite gneiss along ENE-WSW striking thrust, Wadi Abu Ziran, (**b**) Volcanoclastic metasediment thrusted over Abu Fanani schist along S-dipping thrust, Wadi Abu Ziran, (**c**) Dokhan volcanics thrusted over Kujura felsite along SW-dipping thrust, Wadi Queih, (**d**–**f**) Open NW-trending F2 folds in the molasse-type sediments of Wadi Queih.

The earliest S1 in the Meatiq dome is indicated by gneissic banding and schistosity within the amphibolite enclaves of the Um Baanib granite, characterized by a clear orientation of flattened mineral grains with dip angles of 40°- 60°. The E-W fabric appears in the Abu Fanani schist (Figs. 11a and b). The volcanoclastic metasediment and the Abu Fanani schist show southward-dipping thrust imbrications and east-west aligned folds (Fig. 11a and b). These thrusts are folded around N-S oriented folds (Fig. 1c).

The NNW-SSE compression (D1) is evident in the central part of Wadi Queih, where thrust masses of metavolcanics, molasse-type sediments, and felsites form an imbricate thrust belt. Dokhan volcanics are thrust over felsite along a SW-dipping thrust at Wadi Queih (Fig. 11c). The bedding in the molasse-type sediments and the slaty cleavage in the sheared metavolcanics show an east-west orientation, dipping moderately to steeply either northward or southward. In the northeastern basin, ENE-trending folds (F1) are present, characterized mainly by open folds with wavelengths ranging from 1 m to several tens of meters.

#### D2: NW-SE trending structures

The NW-SE-oriented structures are linked to a NE-SW compressional event (D2) and consist of the regional S2 fabric, NW-trending folds, and sinistral shearing along the NW-trending Queih and Abu Ziran shear zones, as well as the associated transpressional imbricate thrusts.

The main tectonic fabric within the Queih shear zone is characterized by widely spaced cleavage oriented NNW-SSE, mainly in sheared metavolcanic and molasse-type sediments. The alternative orientations of slaty cleavage features include ENE-WSW oriented slaty cleavage (S1) preserved in the northeastern part of the molasse-type sediments, NW-SE striking slaty cleavage (S2) mostly found in volcanoclastic metasediments, N-S trending axial planar cleavage (S3) present in the northwestern part of the molasse-type sediments and Dokhan volcanics next to the Um Zarabit granite, and NE-SW trending axial planar cleavage (S4) located in the molasse-type sediments of Wadi Um Zarabit and the metavolcanics of Wadi Himeiriyia.

The thrusting mechanism has caused a significant interleaving of thrust sheets, including metavolcanics, Dokhan volcanic rocks, molasse-type sediments, and felsite. In the central part of the Queih shear zone, the Kujura felsites are thrust over by the Dokhan volcanics (Fig. 11c) and metavolcanics, which sit above the molasse-type sediments, forming an imbricate thrust system. The NNW-dipping thrust marks the boundary between molasse-type sediments and metavolcanics, with bands of mylonitic felsite indicating the thrust contact between Dokhan volcanics and felsite.

The thrusts have undergone distortion and folding due to subsequent geological processes, resulting in orientations shifted to NW-SE and NE-SW, with dips toward the NE and SW at angles ranging from 30° to 65°. Multiple mesoscopic folds are visible in the molasse-type sediments, volcanoclastic metasediments, and sheared metavolcanics. Meanwhile, the regional axial planar structure is characterized by slaty cleavage (S2), oriented northwest with a moderate to steep dip. Additionally, the presence of sigmoidal porphyroclasts in the volcaniclastic metasediments indicates sinistral shearing, and subsequent folding has altered the axial surfaces of the SE-verging folds, leading to reorientation toward E-W, NE-SW, and NNE-SSW. Fine folds directed toward the northwest are clearly outlined along the main foliation and within the arms and joints of the larger folds (Fig. 11d–f).

The Queih shear zone is characterized by four principal regional folds: the Wadi Arak folds, Queih folds, Kujura syncline, Himeiriyia anticline, Abu Zarabit antiform, and Sodmein antiform. The Arak folds have prominent anticlines and synclines in the northeastern region, spanning around 5 to 7 km. These formations consist of molasse-type sediments and Dokhan volcanic materials. The axial traces of these folds change orientation from ENE-WSW to WNW-ESE due to subsequent refolding occurrences.

The Queih folds comprise many NNW-trending anticlines and synclines that meet with the primary course of Wadi Queih (Fig. 11d–f). The folds predominantly orient NNW-SSE, suggesting that the ENE-WSW- and E-W-oriented folds represent older, first-generation folds within the Queih shear zone.

The Kujura syncline is an important fold structure in the central part of the QSB, characterized by an open orientation and a semicircular hinge zone. It has been restructured by NE-SW open folding and includes metavolcanics, molasse-type sediments, Dokhan volcanic rocks, and felsite. The Himeiriyia anticline is a notable geological feature oriented NNW-SSE, situated in the western part of the Queih shear zone. It contains many folded lithologies, such as metavolcanics, molasse-type sediments, and Dokhan volcanics.

**Fig. 12:**
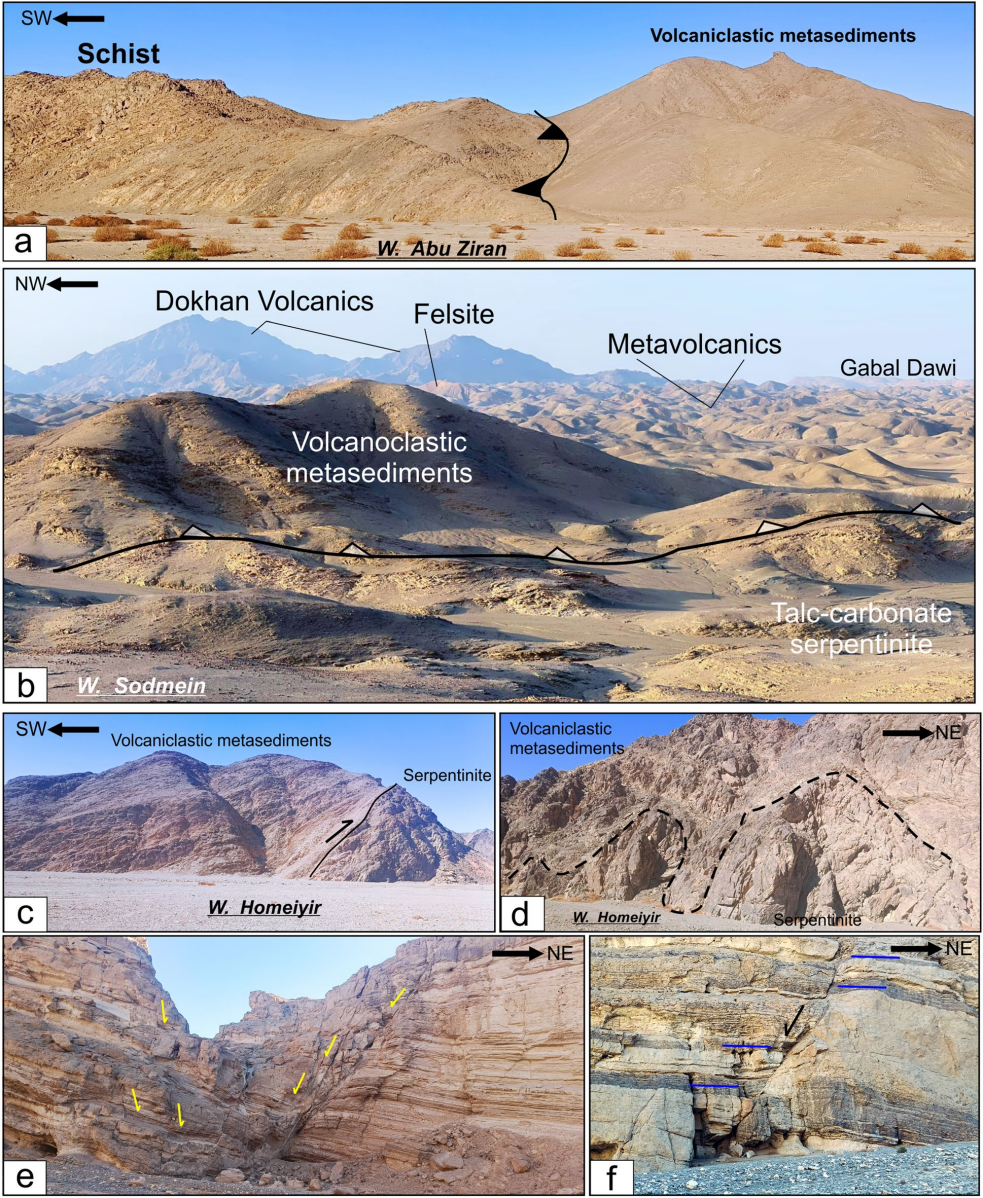
(**a**) Schist and mylonite thrusted over Volcanoclastic metasediment along NE-dipping thrust close to Meatiq gneiss complex, (**b**) general view for Abu Ziran shear zone in Sodmein area showing Volcanoclastic metasediments thrusted over talc-carbonate serpentinite along NE-dipping thrust, (**c**) Volcaniclastic metasediments thrusted over serpentinite Along NE-dipping thrust in Wadi Homeiyir, (**d**) NW-trending folds in volcaniclastic metasediments with fold core containing blocks of serpentinite, Wadi Homeiyir, (**e**) zGraben structure in Dawi Formation (Creatceous) with development of synthetic and antithetic NW-SE striking normal faults. (**f**) NW-trending normal fault in Dawi Formation.

Adjacent to the Meatiq, the region features imbricated thrust sheets that delineate the tectonic boundaries between various thrust sheets within the Abu Ziran shear zone and the neighboring ophiolitic mélange (Fig. 12a). These imbricate thrust faults and ophiolite slices exhibit moderate dips of approximately 30° to 55° toward the northeast.

The Abu Ziran shear zone forms the western tectonic boundary of the Meatiq gneiss complex. It extends about 50 km and reaches a thickness of 6 km, from Wadi Kareim in the southeast to Wadi Sodmein and Wadi Homeiyir in the northwest. The geological makeup along this shear zone includes tectonic mélange, serpentinites, volcaniclastic metasediments, Dokhan volcanics, and felsite. These rocks show significant shearing along thrust planes between serpentinites and volcaniclastic metasediments (Fig. 12b), leading to the development of mylonitic types. The formations are folded around axes trending northwest, creating major anticlines and synclines such as the Haramiya and Abu Ziran folds. F2 folds are visible at different scales, from small outcrops to large formations spanning kilometers. They are generally open and gentle, with axial planes oriented northwest-southeast and fold axes plunging southeastward. These folds include notable anticlines and synclines associated with ophiolitic and volcaniclastic metasedimentary rocks, with fold axes plunging roughly 25° toward N50°W. The axial planes strike N45-55°W and dip 60° to the northeast, aligning closely with the dominant orientation of the foliations (S2) in the folded rocks. Additionally, minor northwest-trending folds are characterized by tight, asymmetric, recumbent, and overturned styles, adding complexity to regional folding. At Wadi Homeiyir, the rocks—comprising volcaniclastic metasediments, serpentinites, and metabasalts—form an imbricate thrust slice oriented northwest-southeast and dipping southwest (Fig. 12c). In Wadi Homeiyir, the fold hinges (Fig. 12d) trend northwest-southeast, parallel to the L2 stretching lineation.

#### N-S trending structures

The N-S structures are connected to an E-W compressional event, characterized by N-S striking axial planar foliation associated with F3 folds. This event involves dextral shearing along the Queih and Abu Ziran shear zones. The prominent twofold plunging anticline forming the Meatiq dome is related to this event and is oriented from north to north-northwest. The N-trending folds (F3) located south of the Meatiq dome, in Wadi Hamariya and between Wadi Abu Zarabit and Wadi Himeiriyia, account for the refolding of both the Kujura syncline and the Himeiriyia anticline (F2). To the south of the Meatiq dome, significant S-dipping thrusts (D1) are twisted around a prominent open N-fold.

During the D3 phase, the original E-W and NW-trending folds, in conjunction with the S-dipping thrusts and NW-trending structures, experienced deformation into N-trending folds as a result of dextral shearing along the NW-SE-oriented Queih and Abu Ziran shear zones. The D3 phase produced N- to NNW-trending tight folds and NNW-SSE thrust-dominated strike-slip shear zones, which were overlaid on pre-existing structures. These shear zones enable the accommodation of auriferous quartz veins and are linked to dextral shearing.

#### D4: NE-SE trending structures

In the D4 phase, dextral shearing within the Dawi shear belt along the Queih and Abu Ziran shear zones caused the folding of earlier structures into many significant NE-trending folds, along with the development of axial planar foliation (S4). Notable folds include the Meatiq Dome, Wadi Hamariya, Wadi Sodmein, and Wadi Homeiyir. The large-scale northeast-trending Wadi Sodmein fold is a syncline that affects all rock units, including the Dokhan volcanics and felsite. Its axes extend roughly 20 km east-northeast. The Kujura syncline (F2) and the Himeiriyia anticline are refolded along the northeast-trending Abu Zarabit and Sodmein antiforms (F4).

#### D5: Phanerozoic structures

This event includes three distinct Phanerozoic deformational periods. The first phase began in the Pre-Cretaceous, leading to the formation of fault-bounded sub-basins. The deposition of the Pre-rift group transformed existing faults into deep-seated faults^116^. The second phase involves the opening of the Red Sea during the Oligocene, reactivating pre-existing faults and resulting in the creation of the Western block. The ongoing opening has formed high terrains and the Red Sea basement belt, establishing the Eastern block and the main rift fault. The proximity of neighboring blocks and displacement along extensional faults (Fig. 12e and f) have enabled the development of folds, mainly synclines (e.g., Al Anz, Hamadat, Atshan, and Nakheil). During the third phase, in the Post-Early Miocene period, additional extensional faulting affected the Eastern block in the Early Miocene epoch, promoting the deposition of the current Syn-rift group deposits^116^. The recent NW–SE normal faults, together with NNW–SSE sinistral strike-slip faults and N–S dextral strike-slip faults, were probably reactivated during the Red Sea rifting.

Abd El-Wahed et al.^117^ identified two significant tectonic events that shaped Phanerozoic rocks: the first involved the creation of Late Cretaceous synclinal basins due to sinistral displacement along the reactivated Najd fault system. The second event was characterized by simultaneous sinistral displacement along the NNE-trending Aqaba–Dead Sea strike-slip fault and dextral movement within the Queih shear zone, resulting in various geological structures, including NW-trending extensional faults (Fig. 12e and f) and buckle folds formed during the Pliocene and post-Pliocene epochs.

**Table 4:** A summary of deformation phases and Key Structures in the Dawi shear belt.

Phase	Structural orientation	Key features and structures	Main lithologies affected	Geological observations
D1 (NNW–SW shortening)	E–W trending	Initial shortening Gneissic banding, schistosity S1 fabric (gneissic banding, schistosity in amphibolite enclaves, Um Baanib granite) Thrust imbrications and folds ENE-trending folds (F1)	Schists, volcaniclastic metasediments, molasse-type sediments, metavolcanics, felsites	Structures poorly preserved, overwritten by later events S1 dip: 40–60° Thrusts folded around N–S folds Open folds with 1 m to 10 s of m wavelengths
D2 (NE–SW compression, NW–SE Najd shearing)	NW–SE trending	S2 regional fabric NW-trending folds, sinistral shearing Imbricate thrusts and major folds (Wadi Arak, Queih, Kujura syncline, Himeiriyia anticline, Abu Zarabit/Sodmein antiforms)	Metavolcanics, molasse-type sediments, Dokhan volcanics, felsite, serpentinites, tectonic mélange	Widely spaced cleavage, slaty cleavage (S1–S4) Major thrusts and mylonitic felsites Fold axes: Plunge ~ 25° toward N50°W Tight/overturned minor folds Four main regional folds; refolding and reorientation of axial traces observed
D3 (E–W compression)	N–S trending	N–S striking axial planar foliation (F3 folds) Dextral shearing along shear zones Tight N–NNW folds, thrust-dominated strike-slip zones	Kujura syncline, Himeiriyia anticline, molasse-type sediments, metavolcanics	Refolding of earlier folds (F2) N–S thrusts contorted around open N-fold Shear zones linked to auriferous quartz veins
D4 (Dextral shearing)	NE–SE trending	NE-trending folds (e.g., Meatiq Dome, Wadi Hamariya, Sodmein, Homeiyir) S4 axial planar foliation Refolding of older structures	Dokhan volcanics, felsite, other regional units	Large-scale synclines, anticlines Axes of folds: ~20 km ENE Refolding along NE-trending antiforms
D5 (Phanerozoic, Red Sea rifting)	Various: NW–SE, NNW–SSE, N–S	Fault-bounded sub-basins Extensional, normal and strike-slip faults Formation of synclines (Al Anz, Hamadat, Atshan, Nakheil)	Phanerozoic rocks, Pre-rift & Syn-rift groups	Reactivation of faults (Najd, Aqaba–Dead Sea) Three deformation periods: Pre-Cretaceous, Oligocene (Red Sea opening), Post-Early Miocene Formation of buckle folds, NW-trending extensional faults

### Fluorite barite occurrences

**Fig. 13:**
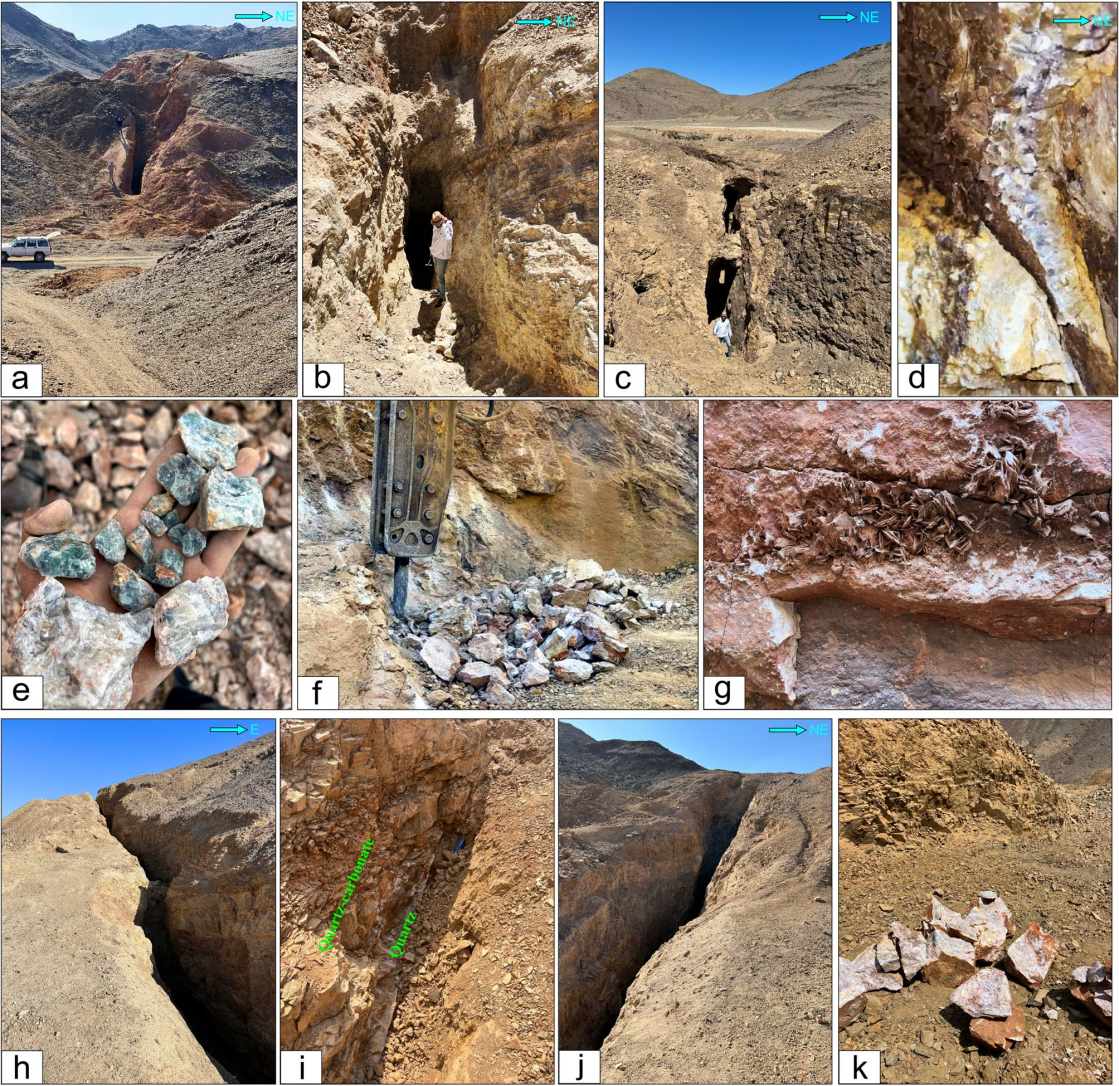
(**a**–**d**) Selective mining in Fluorite vein in Volcaniclastic metasediments in a nearly vertical shear zone, (**e**) and (**f**) white, green and purple fluorite, (**g**) disc-shaped barite crystals (barite rose), (**h**–**j**) Selective extraction within a sheeted NW-trending quartz vein located in a nearly vertical shear zone (approximately 3 m wide). Observe the S-shaped configuration of the narrower quartz veins at the edges of the shear zone, (**k**) Mineralized quartz associated with gold and sulfide minerals like pyrite and arsenopyrite.

Fluorite deposits in Sodmein are found in pegmatitic veins intersecting schists and metavolcanic rocks (Fig. 13a–d). These veins align with regional fault trends that mostly strike NE-SW and NW-SE^118,119^. The fluorite in Sodmein is classified as vein-type mineralization, typically formed by hydrothermal fluids. These fluids have low salinity, indicating that the fluorite was deposited from aqueous solutions at relatively low temperatures^118^. The presence of primary and secondary fluid inclusions within the fluorite crystals offers valuable insights into the formation conditions of these deposits. Chemically, calcium fluoride (CaF₂), known as fluorite, can show various colors due to impurities and formation conditions^119^. In the Sodmein area, white, green, and purple fluorite varieties have been identified (Fig. 13e and f), with differences in their geochemical signatures reflecting the composition of the mineralizing fluids. The fluorite deposits often occur together with other minerals, such as calcite, dolomite, and various sulfides, which are common in hydrothermal vein deposits. This mineral association indicates the geological processes involved in forming these deposits.

Barite (BaSO₄) and fluorite (CaF₂) are two different minerals that often occur together in geological formations. Their co-occurrence is significant in multiple fields, including mineralogy, industrial applications, and metaphysical properties^120^. Barite and fluorite are predominantly located within hydrothermal veins in the Sodmein region, especially disc-shaped barite crystals (barite rose, Fig. 13g). They are frequently associated with other minerals, including malachite, sphalerite, and galena. Both minerals have considerable industrial importance. Barite is primarily used as a weighting agent in drilling fluids for oil and gas exploration. In contrast, fluorite is used in the production of aluminum, gasoline, and uranium fuel, as well as in the fabrication of glass and ceramics^121^.

### Gold occurrences and alteration zones

The gold deposits in the Qift-Quseir region are of considerable economic importance, playing a vital role in Egypt’s mining industry. This area is part of a larger geological structure that encompasses more than 120 identified gold occurrences in the Eastern Desert, making it a crucial site for mineral exploration and extraction.

The Dawi shear belt contains substantial gold deposits associated with mesothermal vein-type occurrences, generally linked to granitic intrusions and hydrothermal processes. Gold mineralization predominantly occurs in granitoid rocks that intrude upon older serpentinites and metabasalts (Fig. 13h–j). The mineralization occurs in quartz veins and is frequently associated with sulfide minerals such as pyrite and arsenopyrite (Fig. 13k), which indicate hydrothermal processes involved in gold deposition^122,123^. The gold deposits are intricately associated with structural elements, including faults and shear zones. These structures enable the circulation of hydrothermal fluids abundant in gold and other minerals. The primary structural orientations in the region are NW-SE and NE-SW, which are essential for understanding the distribution of gold mineralization^124,125^. The wall-rock alteration adjacent to the gold-bearing quartz veins displays many alteration zones, including phyllic and propylitic changes. These modifications come from hydrothermal fluids interacting with the host rocks, culminating in the concentration of gold in particular regions. Research indicates that the gold content within mineralized zones can vary considerably, with specific areas exhibiting elevated gold concentrations associated with sulfide minerals^122,123^. Recent exploration initiatives in the Qift-Quseir region have employed geophysical techniques, such as aeromagnetic and aeroradiometric surveys, to identify prospective gold mineralization areas. These methodologies assist in identifying structural characteristics and alteration zones that are conducive to gold resources^124,125^.

Gold mineralization in Sodmein and El Haramiya is intricately associated with hydrothermal alteration processes. These processes involve the circulation of mineral-laden fluids that modify the surrounding rocks, thereby concentrating gold in specific areas. Common alteration types include phyllic and propylitic modifications, which indicate the conditions under which gold was deposited^21,124^. Recent exploratory assessments have discovered multiple zones of gold mineralization within these regions (Fig. 13h–j). The results indicate that gold frequently occurs alongside sulfide minerals such as pyrite and arsenopyrite, suggesting the presence of gold-bearing quartz veins^21,126^. Research indicates that gold richness in these regions can vary, with some sites exhibiting substantial concentrations. Quartz and quartz-carbonate veins (Fig. 13i) are particularly significant, since they frequently serve as the principal hosts for gold mineralization^21,124,126^.

The El Haramiya–Sodmein gold occurrence includes ancient placer workings, dwellings, crushing milestones, and ore-washing pits. The region has multiple mineralizations, including antigorite serpentinite, which has lower Au, As, and S due to mantle-derived CO_2_ during carbonate alteration^21^. El Shimi, et al.^127^ reported values from 0.36 to 1.53 ppm, averaging 0.945 ppm. Abdelfadil et al.^25^ suggested listevinite transitions into serpentinized peridotites during listevinitization, reducing magnetic susceptibility compared to serpentinites.

The ancient Egyptians exploited quartz veins and placer deposits in the El Haramiya–Sodmein gold occurrence area. Here, signs of placer mining, remnants of ancient habitations, millstones used for grinding, and ore-washing pits have been found in the higher regions of Wadi El Haramiya. This area contains six small gold placer occurrences situated along ancient terraces across four minor wadis, with quartz vein fragments inclined at 50–80° NE, reaching lengths of up to 400 m and thicknesses of 0.3–0.8 m^21^. These veins are predominantly composed of large quartz formations interspersed with pyrite, although the precise gold content within these auriferous veins has yet to be established^127^.

One to three free gold particles with dendritic and lamellar morphologies, ranging in size from 0.1 to 1.0 mm, were found in exploratory surveys carried out by Egypt and Russia in 1973–1974. Bedrock sampling revealed gold concentrations ranging from 0.003 to 0.6 g/t, with the highest concentrations found in quartz and quartz-carbonate veins, as well as adjacent schists. The highest concentration was found in a granite porphyry formation that resembled a dyke^21,127^ (Table 5).

**Table 5:** Comparative summary of fluorite, barite, and gold occurrences and characteristics in the Dawi shear belt, Egypt.

Feature/aspect	Fluorite	Barite	Gold
Primary locality	Sodmein	Sodmein	Qift-Quseir region, Dawi shear belt, Sodmein, El Haramiya
Host rock/setting	Pegmatitic veins in schists & metavolcanics	Hydrothermal veins with fluorite	Granitoid intrusions in serpentinites/metabasalts, quartz veins, schists, and placer deposits
Structural controls	Veins align NE-SW, NW-SE faults	Hydrothermal veins, disc-shaped crystals	Controlled by faults and shear zones (NW-SE & NE-SW orientations)
Mineralization style	Vein-type, hydrothermal, low salinity fluids	Hydrothermal, often with fluorite	Mesothermal vein-type, hydrothermal, structurally controlled
Colors/varieties	White, green, purple fluorite	Barite rose (disc-shaped), white/colorless	Free gold (dendritic, lamellar); quartz and quartz-carbonate veins
Associated minerals	Calcite, dolomite, sulfides	Fluorite, malachite, sphalerite, galena	Pyrite, arsenopyrite, quartz, quartz-carbonate, serpentine, placer gold
Key alteration	-	-	Phyllic, propylitic, listevinite, carbonate alteration
Formation conditions	Low temperature, hydrothermal fluids	Hydrothermal, co-precipitation with fluorite	Hydrothermal fluids, alteration zones, circulation via faults/shear zones
Economic uses	Aluminum, gasoline, uranium fuel, glass, ceramics	Drilling fluids for oil & gas	Mining resource: economic importance for Egypt’s gold industry
Industrial importance	High	High	Very high
Exploration methods	–	–	Geophysical (aeromagnetic, aeroradiometric) surveys
Historical evidence	–	–	Ancient placer mining, habitation remains, millstones, and ore-washing pits.
Grades/concentration	–	–	0.36–1.53 ppm (avg. 0.945 ppm); 0.003–0.6 g/t bedrock; placer gold particles 0.1–1 mm
References	^118^^119^;	^120^^121^;	^4^^31^^57^^21^^122^^123^^124^^125^^126^^127^;;;;;;;;

## Discussion

### Lithological mapping and structural analysis utilizing Landsat-8

High-resolution color-composite analysis in the Egyptian Eastern Desert is effective for lithological and structural mapping, as demonstrated by various authors^6,7,10,40,47,49,50,128–130^. Enhanced Landsat-8 images, including Fcc765, decorrelated 765, band ratios (BRs) 2/5, 3/6, 3/7, 6/7, 4/3, 5/4, and principal components (PCs) 413 and 214 in RGB mode, effectively distinguish lithological units and boundaries. These methods helped trace valleys, verify rock units and their distribution, and contributed to producing an accurate geological map (Fig. 1c). They also identified mylonitic zones in Umm Ba’anib gneiss (Figs. 2c and d and 3b, and 3c), as well as magmatic intrusions such as syn- and late-tectonic granites and parts of syn-tectonic metagabbro (Fig. 3a, b). The complete sedimentary sequence from Cretaceous to Quaternary periods was visible in most images. Clearer boundaries among ophiolitic and arc assemblages appeared with decorrelated 765 and certain BRs (Fig. 2b–d).

The pan-sharpened imagery, along with the apparent variation in the rock colors, allowed monitoring and tracing the offset and bending of the lithological contacts, aiding in identifying the significant structural elements (e.g., faults, folds, foliations) presented in the examined area, utilizing the remotely sensed colored imagery of Landsat-8 (Figs. 2a and 4a and c). The obtained structural elements and their spatial distribution enabled us to divide the study area into three domains: northeastern, southwestern, and southeastern, according to the intensity of the structural elements in each domain (Fig. 2a).

The first domain (Domain I) is exposed in the northeastern sector west of W. Queih (Figs. 2a and 4a and b), characterized by folded arc assemblages (e.g., metavolcanic and volcanoclastic metasediments), felsite, Dokhan volcanics, and molasse-conglomerate compositions, as well as some late-granitic intrusions in the extremely northern part of this province. This area is dissected and dominated by several major and minor sinistral strike-slip faults, mainly NW to NNW, along with two other groups of major dextral strike-slip faults, which are primarily oriented N-S, NE, ENE, and E-W (Fig. 4b). Additionally, this region features a set of folded layers with NW-axial planes and NW-thrust contacts, which postdate the recorded strike-slip faults and are separated between the arc assemblages, felsite, Dokhan volcanic, and molasse-conglomerate. Based on the observed cross-cutting relationships among the identified strike-slip faults, thrusts, and fold axes, it can be concluded that the earliest strike-slip group consists of the NW-sinistral faults related to the NW-Najd fault system (NFS), followed by the dextral N-S faults associated with the first outlined N-S shear belt defined^10^, and finally, the dextral ENE to E-W faults linked to the extension phase affecting the EED.

The highest seconded deformed domain (Domain II), which occurs in the southwestern sector along W. Abu Ziran, is characterized by gneissic, ophiolitic, arc assemblages, and gabbroic to granitic intrusion units, as well as the presence of mylonitic zones (mylonitic gneiss and schist). These zones are separated from the gneissic rocks by a series of NW-SE to N-S thrust faults (Figs. 2a and 5c and d). This fault set is cut by several minor sinistral strike-slip faults trending NW (Fig. 4d). A dominant NW-thrust fault set separates the ophiolitic units from the arc units, which are dissected by several dextral strike-slip faults striking mainly NE and oriented N-S in the central part of the province. Folded foliations with NW to NNW-axial planes are more prominent in the ophiolitic mélange at the extreme southern part, which is cut by a major NW-sinistral strike-slip fault associated with NFS (Figs. 4c and d). As previously mentioned, cross-cutting relations reveal that the developed folds and thrusts predate the NW strike-slip faults, which are followed by NE and N-S dextral strike-slip faults.

For the third weak domain (Domain III) located in the southeastern sector west of W. Kareim, it is dominated by a set of NW-normal faults that separate the arc, Cretaceous, Tertiary, and Quaternary units, followed and dissected by two major dextral strike-slip faults striking E-W (Fig. 2a). Lithologically, this sector is primarily composed of sedimentary sequences along with parts of volcanoclastic metasediments, Dokhan volcanics, and late-granite intrusions, which form the core of the area. The dominance of these sedimentary sequences explains the weakness of the deformation.

### Optical and SAR data integration for the mineralization mapping

The integration of various data types, including lithological, structural, and remote-sensing information, is regarded as one of the most effective approaches for identifying high-potential mineralized zones. In the context of targeting high-potential zones of gold, fluorite, and other ore minerals, an integration of optical, SAR data, and field data—demonstrated as highly effective in previous studies by researchers^7,10,11,31,40,44,47,48^ has been used to highlight and map these zones. It is widely accepted that the main criteria for ore exploration, such as gold and fluorite, include host lithology, structural features, and hydrothermal alteration zones. Therefore, identifying these hydrothermal alteration zones, which are favorable for ore mineralization, is a key element in exploring ore areas.

**Fig. 14:**
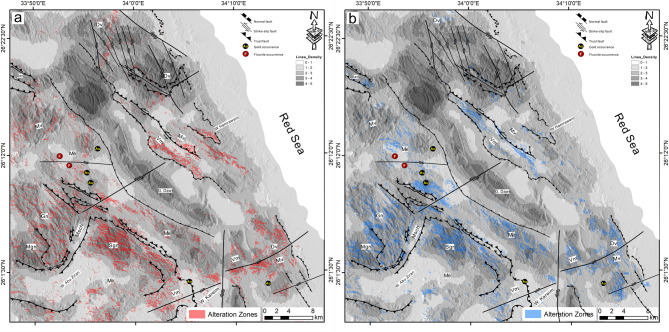
Lineament Density vs. the spatial distribution of alteration zones, structural elements, lithological units and ore occurrences by (a) ASTER and (b) Landsat-8. For abbreviation see Fig. 1. (Created by ArcGIS Desktop 10.8. (https://www.esri.com/en-us/arcgis/products/arcgis-desktop/overview/).

High lineament and fracture density signifies extensive rock fracturing, linked to increased gold mineralization potential. The lineament density map from S1A radar data served as a base to evaluate rock units, structures, and alteration zones identified from Landsat-8 and ASTER imagery (Fig. 14a, b). Most alteration zones, especially high-density ones, overlap with areas of moderate to high lineament density featuring ophiolite slices, metavolcanics, and syn-tectonic granite (Fig. 14a, b). Comparison between alteration zones detected by BRs and SAM methods on Landsat-8 and ASTER also showed coinciding sites, aligned with high-density alteration zones (Figs. 2c and d and 4a, c and f). Gold and fluorite occurrences further match these alteration zones (Fig. 14). This overlap confirms the accuracy of the alteration zones, verified by fieldwork.

**Fig. 15:**
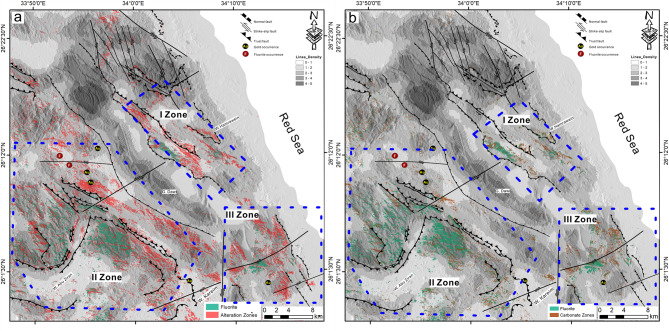
(**a**) and (**b**) Potential maps for gold and fluorite exploration using S1A radar and optical data. These figures were created and processed by ENVI v. 5.6.2. software: https://www.l3harrisgeospatial.com/Software-Technology/ENVI), which is mainly utilized for image processing, and ArcGIS Desktop 10.8. (https://www.esri.com/en-us/arcgis/products/arcgis-desktop/overview/).

Previous research shows that fluorite ore is mainly distributed near the contact zone between carbonate rocks and metamorphic rocks^131,132^. So, another integration has been conducted between the aforementioned key elements, fluorite occurrences and the surface distribution of carbonate minerals (e.g., calcite, dolomite, magnesite) which were utilized as base map for the fluorite surface distribution, revealing the correlation between the fluorite zones and moderately to high lineament density areas marked by dense alterations and carbonate zones located at I, II and III Zones (Fig. 15a and b).

The integrated maps (Figs. 14 and 15) demonstrate that NW-striking lineaments aligned with the NFS play a vital role in controlling the distribution and concentration of alteration minerals and related ores, such as gold and fluorite, which are primarily sourced from hydrothermal fluids ascending through host rocks with high lineament density (e.g., ophiolitic mélange, metavolcanic, volcanoclastic metasediment, syn-granite). The overlap of the fluorite zones with the alteration and carbonate zones indicates a strong association between fluorite minerals and carbonate areas. It was revealed that hydrothermal fluids are a primary source of fluorite in the study area. As a result of the integration, the study areas were subdivided into three potential ore exploration zones, where ore minerals such as gold and fluorite are likely to occur. These zones are: (1) I Zone, southwest G. Dawi along W. Abu Ziran, (2) II Zone, west of W. Kareim at the southeastern part, and (3) III Zone, along W. Hamrawein at the eastern part of the study area (Fig. 15).

### Subsurface basement structures in the Dawi shear belt

The integration of SAR and aeromagnetic datasets was employed to leverage their complementary strengths: SAR captures surface geomorphological expressions related to structural fabrics, while aeromagnetic data reveals subsurface structural discontinuities and deep-seated tectonic controls. This multi-dataset approach enabled comprehensive characterization of lineaments from surface to depth. The strong spatial coincidence between lineaments extracted from both datasets serves as internal validation of their geological significance, allowing confident interpretation of genuine structural features while minimizing the risk of including non-geological artifacts.

The use of airborne geophysical data is an efficient and robust tool for analyzing subsurface patterns, especially in structurally complex regions. In this study, the EHGA approach applied to RTP data uncovered a series of common shallow structural directions, including NW, NE, NNE, N–S, ENE, and WNW. These trends influence the shallow geological structure and align with the known tectonic setting in the Dawi shear belt area. At depths of 0.5, 1, and 2 km, the structural framework becomes simpler, showing NW, ENE, and NE as the dominant orientations. The results from EHGA (Fig. 8) and Eu-D (Fig. 9) indicate that NW is the prevailing trend in the region. This vertical variation reveals the plunging crustal architecture and the continuation of major fault patterns. The reliability of the EHGA results was confirmed by Eu-D and SPI, which yielded similar structural directions and depth estimates up to approximately 2.7–2.9 km (Figs. 8, 9 and 10). Notably, several structural features identified at shallow depths disappear with increasing depth and are supported by consistent shallow Eu-D solutions (Fig. 9), confirming that these features are shallow components. Combining multiple methods offers a comprehensive understanding of both shallow and deep structural controls, which can assist mineral exploration efforts.

Comparing our findings with previous investigations, the strength of this strategy becomes prominent. For example^21^, used SPI and Euler methods in the El Haramiya and Sodmein regions and emphasized NW and NE systems as prevailing controls on listvenite-altered zones and gold-bearing-quartz veins^21^. While they demonstrated the effect of shear zones in mineral deposits, their analysis focused on near-surface structural elements and profile-based examination. In contrast, the current study uses full-region boundary detection and depth-based filtering to characterize continued deep-seated structural lineaments and localized surface components, providing a more precise order of structural controls. Likewise, Hegab^133^ depended on radiometric and remote sensing data to outline uranium enrichment and hydrothermal alterations in the Duwi region but lacked the structural solution at depths that aeromagnetic data introduced. Ghoneim et al.^62^ used machine learning and remote sensing for lithological mapping, acquiring high surface mapping precision but without understanding the continuity of structural lineaments downward. By integrating surface and shallow structural mapping from remote sensing analysis and field studies with depth solutions obtained from EHGA, SPI, and Eu-D, our present analysis presents a comprehensive model for determining potential zones for mineralization, mainly where alteration zones or surface anomalies intersect with deep-seated structural networks, a crucial step for prospective exploration targeting gold, fluorite, and barite in comparable environments.

### Structure control of gold and fluorite in the Dawi shear belt

The distribution and localization of gold and fluorite mineralization within the Dawi shear belt are strongly influenced by structural features that have facilitated ore deposition. The Dawi shear belt is characterized by a network of ductile to brittle-ductile shear zones that have served as primary conduits for hydrothermal fluid migration and subsequent mineral precipitation^134,135^.

The Dawi shear belt experienced a sequence of deformation phases, beginning with NNW-SW shortening (D1), followed by ENE-WSW compression and sinistral transpression due to Najd shearing (D2), then prolonged E-W compression forming N-S folds and dextral shearing (D3, subsequent NE-trending folds (D4), and finally, reactivation of multiple fault types during the Cretaceous and Red Sea rifting (D5).

The predominant tectonic fabric in the Queih shear zone area is characterized by widely spaced NNW-SSE-oriented cleavage, which is mainly found in sheared metavolcanic and molasse-type sediments. The thrusting mechanism has resulted in considerable interleaving of thrust sheets comprising metavolcanics, Dokhan volcanics, molasse-type sediments, and felsite. The Meatiq region is distinguished by imbricated thrust sheets that delineate the tectonic boundaries within the Abu Ziran shear zone and the surrounding ophiolitic mélange. The geological makeup along the Abu Ziran shear zone comprises serpentinites, volcaniclastic metasediments, Dokhan volcanics, and felsite, which exhibit pronounced shearing along thrust planes, resulting in mylonitic varieties.

The N-S trending structures are associated with an E-W compressional event, featuring N-S striking axial planar foliation linked to F3 folds. During the D4 phase, dextral shearing within the Dawi shear belt folded earlier structures into numerous significant NE-trending folds and developed axial-planar foliation.

Fluorite mineralization is closely linked to structural controls within the shear belt. Fluorite occurs both as discrete veinlets and as disseminations within hydrothermal quartz-barite veins that occupy extensional fractures and brecciated zones along the shear belt. The spatial association of fluorite with gold-bearing veins suggests a genetic relationship, likely reflecting episodic fluid flow along shear zones^136^. Fluorite deposits in Sodmein occur in pegmatitic veins that intersect schists and metavolcanic rocks and are classified as vein-type mineralization formed by hydrothermal fluids. These pegmatitic veins align with regional fault trends that predominantly strike NE-SW and NW-SE. These directions are created as tension fractures associated with the intrusion of late tectonic granites.

Gold mineralization within the Dawi shear belt is predominantly hosted by granitoid rocks that intrude older serpentinites and metabasalts and is frequently associated with sulfide minerals such as pyrite and arsenopyrite. Field observations indicate that gold-bearing veins are commonly associated with second-order splays and dilational jogs formed during transpressional deformation, which collectively enhance the fluid flow and mineral deposition^137^.

Structural features, including faults and shear zones, facilitate the movement of hydrothermal fluids. These shear zones are primarily characterized by a NW-trending thrust-dominated transpressional zone related to the Najd Fault System.

In the Sodmein and El Haramiya regions, gold mineralization is linked to hydrothermal alteration, with phyllic alteration and propylitic changes indicating the environmental conditions during gold deposition. Recent exploration has identified numerous gold mineralization zones in these areas, especially noting quartz and quartz-carbonate veins..

The structural architecture of the Dawi shear belt, including high-strain zones, fault jogs, and extensional sites, has played a critical role in controlling the localization, geometry, and grade of both gold and fluorite mineralization. These findings align with models of structurally-controlled epithermal and orogenic gold systems reported in similar tectonic settings worldwide^134,135^.

## Conclusions

This study demonstrates that integrating multispectral remote sensing, SAR, and aeromagnetic data is highly effective for unraveling the complex structural controls and mineralization patterns within the Dawi shear belt in Egypt. Integrated geological and geophysical analysis successfully delineated the main lithological units, major and subsidiary structural features, and hydrothermal alteration zones hosting gold, fluorite, and barite mineralization. The combined use of Landsat-8, ASTER, Sentinel-1 A SAR, and aeromagnetic data provided complementary perspectives, allowing for robust mapping of both surface and subsurface structures.

Structural mapping revealed that NW-SE-, NE-SW-, and N-S-trending shear zones, faults, and folds serve as the main conduits for hydrothermal fluids, controlling the localization and geometry of mineralized zones. The deformation history, dominated by polyphase shearing and folding, has created favorable settings for ore deposition, particularly along the Najd Fault System and its associated structures.

The RTP aeromagnetic data were used to map deep-seated and shallow structures in the Dawi shear belt. The EHGA filter was applied to RTP and UWC data at altitudes of 0.5, 1, and 2 km. The results of EHGA revealed that NW, NE, ENE, NNW to N-S, and WNW are the main shallow lineaments, while the NW, ENE, and NE are the deep-seated prevailing structures. The SPI and Eu-D were used to map depths of structures and basement topography. The obtained depth results are about 2.7 km for Eu-D and 2.9 km for SPI.

Hydrothermal alteration mapping identified three prospective zones with overlapping high lineament density, alteration mineral assemblages, and carbonate occurrences. These zones correspond to the most favorable targets for gold and fluorite exploration, confirming the effectiveness of integrated remote sensing and geophysical workflows.

Fluorite deposits in Sodmein are found within pegmatitic veins that cut through schists and metavolcanic rocks, representing vein-type mineralization formed by hydrothermal fluids of low salinity at relatively low temperatures; these deposits feature fluorite in various colors—white, green, and purple—and are commonly associated with calcite, dolomite, and sulfide minerals. Gold mineralization is predominantly associated with quartz and quartz-carbonate veins within granitoid and metavolcanic host rocks, closely linked to NW- and NE-trending shear structures. Fluorite and barite occur mainly within hydrothermal veins aligned with regional fault systems, particularly where carbonate and pegmatitic rocks are intersected by shearing.

The multi-sensor approach employed in this study offers a transferable framework for targeting mineralization in similar Precambrian terranes worldwide. By correlating surface alteration, structural complexity, and subsurface geophysical signatures, exploration efforts can be more accurately focused on the highest-potential zones.

## Data Availability

Data sets generated during the current study are available from the corresponding author on reasonable request.
